# DNA Volume, Topology, and Flexibility Dictate Nanopore Current Signals

**DOI:** 10.1021/acs.nanolett.3c01823

**Published:** 2023-07-24

**Authors:** Yunxuan Li, Sarah E. Sandler, Ulrich F. Keyser, Jinbo Zhu

**Affiliations:** † Cavendish Laboratory, University of Cambridge, JJ Thompson Avenue, Cambridge CB3 0HE, United Kingdom; ‡ School of Biomedical Engineering, Faculty of Medicine, Dalian University of Technology, No. 2 Linggong Road, Dalian 116024, China

**Keywords:** nanopore sensing, DNA properties, circular DNA

## Abstract

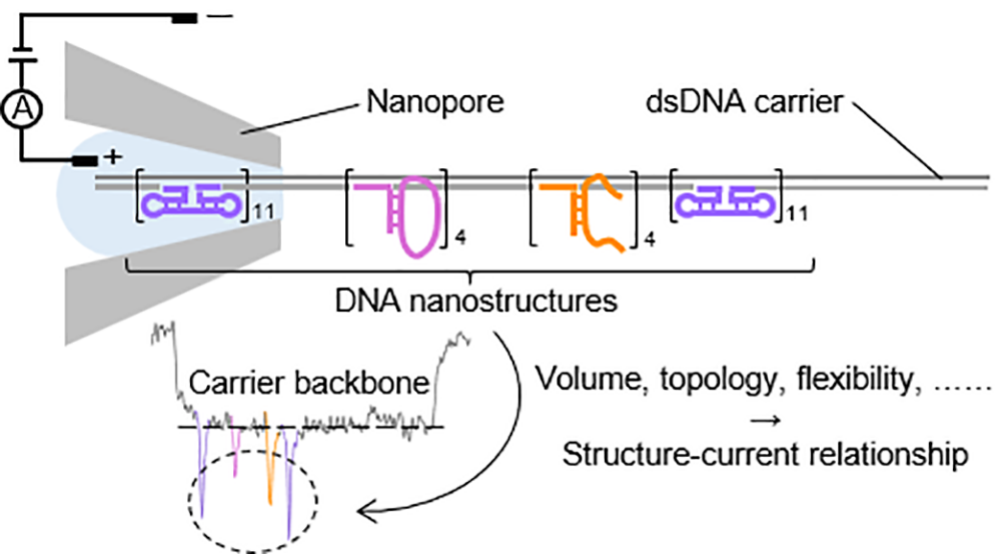

Nanopores have developed into powerful single-molecule sensors capable of identifying and characterizing small polymers, such as DNA, by electrophoretically driving them through a nanoscale pore and monitoring temporary blockades in the ionic pore current. However, the relationship between nanopore signals and the physical properties of DNA remains only partly understood. Herein, we introduce a programmable DNA carrier platform to capture carefully designed DNA nanostructures. Controlled translocation experiments through our glass nanopores allowed us to disentangle this relationship. We vary DNA topology by changing the length, strand duplications, sequence, unpaired nucleotides, and rigidity of the analyte DNA and find that the ionic current drop is mainly determined by the volume and flexibility of the DNA nanostructure in the nanopore. Finally, we use our understanding of the role of DNA topology to discriminate circular single-stranded DNA molecules from linear ones with the same number of nucleotides using the nanopore signal.

Ever since the introduction of resistive pulse sensing and patch clamping techniques, nanopores have drawn wide attention as versatile and efficient platforms for rapid single-molecule sensing without the need for labeling, enzyme reactions, and amplification steps that may introduce biases. In particular, solid-state nanopores have overcome some limitations of traditional nanopore setups in recent years, allowing variation of pore size/geometry, adaptability to a broad range of conditions (i.e., pH, temperature, and ionic concentration), and compatibility with electronic and optical readout systems.^1−6^ So far, a variety of solid-state nanopore fabrication methods have been explored,^7−14^ among which laser-assisted pulling of glass capillaries has stood out for its relatively low cost and substantial ease of operation. Typically, needle-shaped nanopores are formed by applying heat at a central spot of a glass capillary and a pulling force at both capillary ends. They are ideally suited for the analysis of double-stranded DNA (dsDNA) with lengths of several kilobases (kb) and have proved successful in detecting diverse DNA nanostructures, including DNA tetrahedrons,^15,16^ multiarm DNA concatemers,^17,18^ concentric square DNA origami,^19,20^ and many more.

In particular, an effective way to detect DNA structures using glass nanopores is to design a DNA strand (named the DNA carrier) to which target molecules can be attached. Even small analytes can induce a distinct secondary peak superimposed on the ionic current blockade generated by the carrier alone and thus can be easily distinguished. Also, the positions of staple oligonucleotides can be controlled with remarkable ease, making it possible to detect multiple targets concurrently. In previous work, DNA nanostructures such as dumbbells,^21,22^ multiway DNA junctions,^23^ G-quadruplex,^24,25^ and hairpins^26^ have been successfully immobilized on DNA carriers and detected by nanopores. However, how the additional DNA structures regulate nanopore current signals is not yet fully understood. In this study, we introduce a new carrier design with two references and four functional positions to establish the structure–current relationship in nanopores. Additionally, we use our DNA carrier sensor to distinguish ring-shaped single-stranded DNA (ssDNA) molecules from their linear counterparts. Considering the characteristic geometrical and topological properties of circular DNA, as well as the crucial role that DNA cyclization plays in the formation and treatment of viruses and cancers,^27−29^ this work demonstrates that the use of nanopore platforms for single-molecule sensing can be expanded to aid in the development of the clinical applications of the nanopore technology.

## Design of DNA Carrier Platform

**Figure 1:**
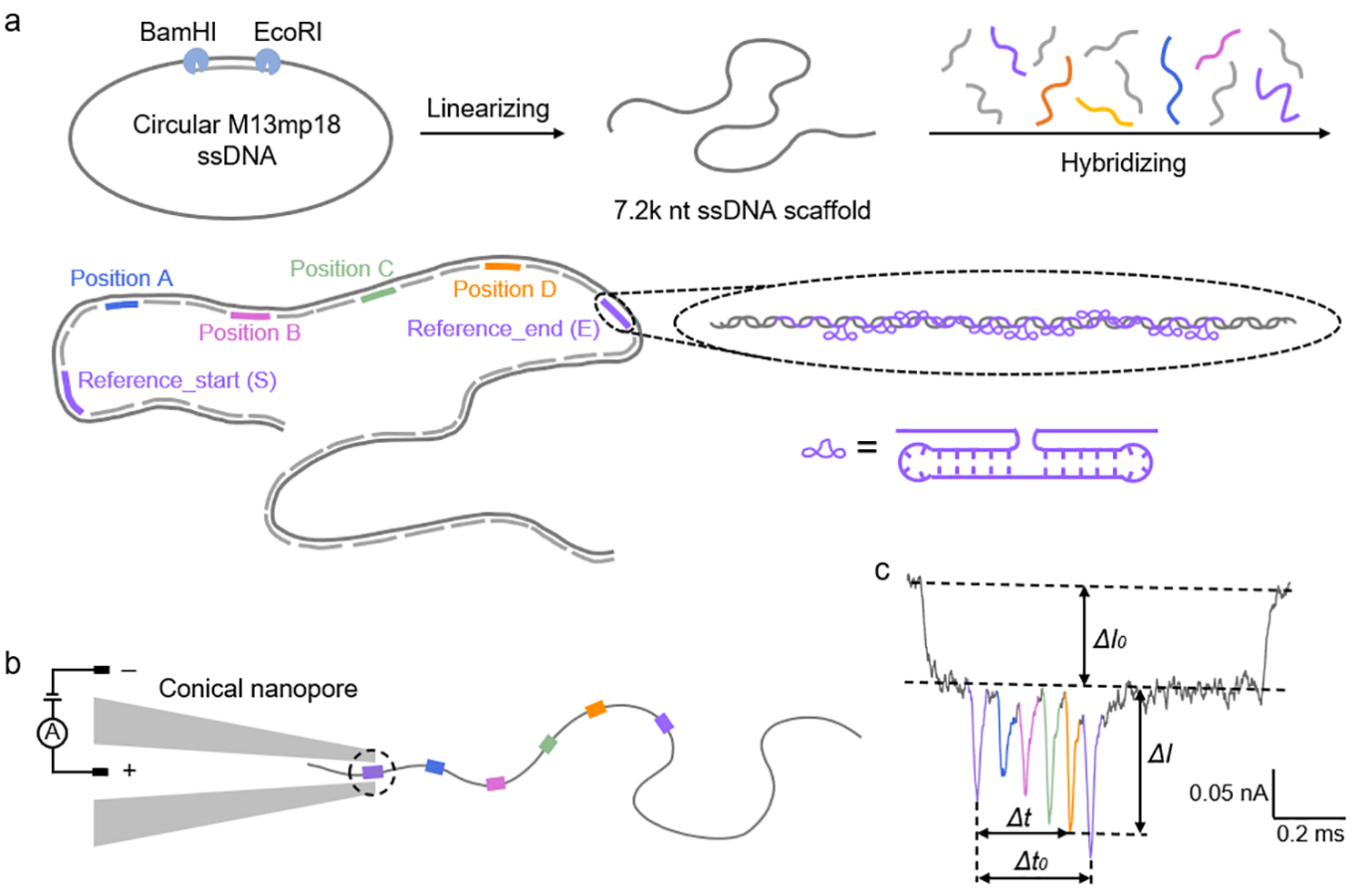
Preparation and detection of DNA carriers. (a) Schematic of the preparation of DNA carriers from circular M13mp18 ssDNA by cutting at the *Bam*HI and *Eco*RI restriction sites and then hybridizing with complementary oligonucleotides. Target DNA nanostructures can be attached to well-defined positions along the contour by replacing individual oligonucleotides. Different functional positions are indicated by different colors. Purple indicates reference structures (the one at the end of the carrier is called “reference_start” (S), and the one in the middle part is called “reference_end” (E)); blue indicates position A; pink indicates position B; green indicates position C; and orange indicates position D. (b) Schematic of a carrier passing through a nanopore under the application of an electric potential. The references and binding sites A–D are shown as colored rectangles, while the long dsDNA is shown as a continuous gray line. The black-dashed circle highlights the sensitive region of the nanopore tip that determine the generation and change of ionic current signals. (c) Current signal of a representative unfolded event. It is defined as Peakdepth = Δ*I*/Δ*I*_0_ and Location = Δ*t*/Δ*t*_0_, where Δ*I*_0_ is the average of the first-level current drop, Δ*I* is the secondary current change beyond the first-level plateau, Δ*t*_0_ is the time scale between the two peaks generated by reference structures, and Δ*t* is the interval between any target peak and the peak suggesting reference S.

As illustrated in Figure 1a, we hybridize short DNA oligonucleotides to the linearized ssDNA of the M13 bacteriophage to create a long dsDNA carrier. As we know the sequence of each oligonucleotide, we can design DNA molecules that contain distinct patterns. The asymmetric design of dumbbell references, which are labeled as S and E for the start and the end of the carrier, respectively, allows us to identify the direction in which the carrier flows through the nanopore. All current data of the references will be shown in purple. Between S and E, we define four functional positions labeled A (blue), B (pink), C (green), and D (orange). We immobilize DNA strands of different numbers of duplications, lengths, sequences, and shapes at the functional positions for direct comparison in the same nanopore. Detailed sequences of the dumbbells and all of the other customized short oligonucleotides can be found in Tables S2–7. These structures will lead to a large DNA blockade signal that is easily detected in our nanopore sensors, as explained in Supporting Information S2.1. It is important to note that the illustration is not drawn to scale, and between each binding sites there are 11 oligonucleotides to achieve an average distance of 418 base pairs (bp).

A sketch of our nanopore setup with a DNA carrier at the start of the translocation is shown in Figure 1b. Figure 1c displays a typical event of a DNA carrier passing through the nanopore, driven by an electric field. The trace shows the ionic current through the nanopore as a function of time with six peaks for the two references and four target structures. The colors on the spikes correspond to the colors of structures on the carrier in Figure 1a,b. We tested the influence of attachment position on the nanopore current signal and found that it is negligible compared to the difference brought about by different target structures on the same carrier (Supporting Information S3.1). As we are using nanopores with ∼10 nm diameters in 4 M LiCl solution, the design and signal-to-noise ratio allow for data analysis using simple thresholding.

## Dependence on the Number and Length of DNA Overhangs

**Figure 2:**
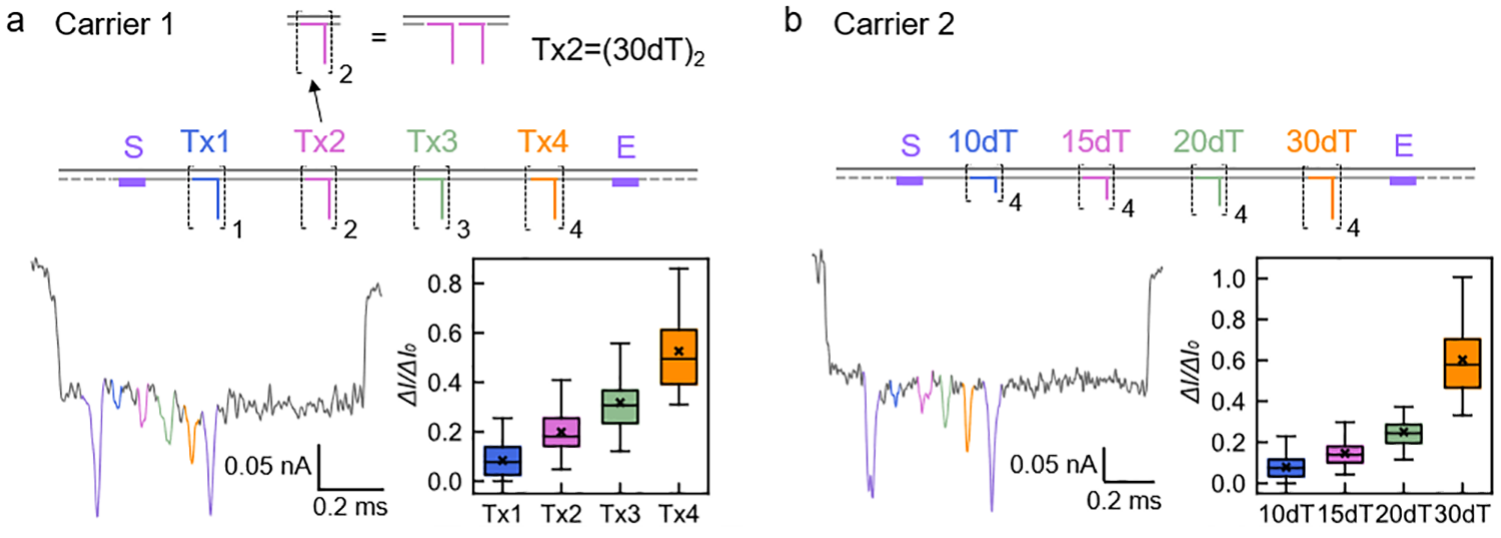
Studies on the dependence of the nanopore current signal on the number and length of DNA overhangs. All the statistical boxplots in this paper are based on a normalized analysis of the first 100 unfolded events recorded in each experiment. The average peak depths are marked by × in the boxplots, while the median peak depths are labeled by horizontal lines. (a) Schematic and nanopore measurement result of Carrier 1 with different numbers of poly dT overhangs. “Tx1” to “Tx4” refer to 30 nt poly dT overhangs with one to four duplications, respectively. (b) Schematic and nanopore measurement result of Carrier 2 with poly dT overhangs of different lengths. “10dT” to “30dT” refer to poly dT overhangs with a length of 10 to 30 nt. Except for Carrier 1, there are four repeats in each structure on all the other carriers.

We started by investigating the influence of the number and length of ssDNA overhangs on the current signal. A previous study has shown that a single short oligonucleotide on a DNA carrier cannot generate observable secondary current drop in a nanopore with a 10 nm diameter.^23^ However, duplication of other small DNA nanostructures, like dumbbell hairpins, significantly enhances the nanopore current signal.^22^ Therefore, we decided to study how the increase in the number of ssDNA overhangs makes them discernible by our nanopore sensor. Since poly(deoxythymidine) (poly dT) is an excellent ssDNA free from any secondary structure,^30−32^ we used staple strands all with poly dT overhangs here. The design is shown at the top of Figure 2a with Carrier 1 having 1, 2, 3, and 4 poly dT strands at binding sites A–D, respectively. Detailed structures of all of the carriers designed in this work can be found in Supporting Figures S1–4. It is obvious that the single strand at position A is not enough to produce a clear signal. Increasing the number of strands to 2 and 3 in Figure 2a suggests that two or three repeats of 30 nucleotides (nt) poly dT are enough for producing an identifiable small peak superimposed on the first-level current drop. However, we find that four repeats at position D generate the most distinct resistive pulse on average. In the boxplot, the ratio of the secondary current drop normalized at the dsDNA level, Δ*I*/Δ*I*_0_, clearly shows the expected trend to larger resistive spikes.

We then studied whether shorter overhangs than 30 nt with four repeats can also result in obvious current changes. Positions A–D had 10, 15, 20, and 30 nt, respectively, in Carrier 2. As shown in Figure 2b, it is quite difficult to discriminate 10 nt overhangs from the background noise in most cases. Although a large portion of events show discernible peaks at position B for 15 nt overhangs, there are still some events with peaks that are missed. The 20 nt overhangs at position C are detectable in a majority of the events, and hence, we decide that 20 nt is the smallest length of four-repeated poly dTs for robust detection in our system. And as expected, four repeats of 30 nt are easy to detect. The relationship of Δ*I*/Δ*I*_0_ and the number and length of poly dT overhangs is more clearly explained in the fittings shown in Supporting Information S2.2. We also proved that an increase in the length of an overhang does not result in more significant nanopore signals than an increase in the repeats of the overhang to reach the same number of nucleotides (Supporting Information S3.2). Therefore, we decided to take the strategy of having four repeats in each structure on the following carriers to enhance nanopore signal.

## Dependence on DNA Sequence

**Figure 3:**
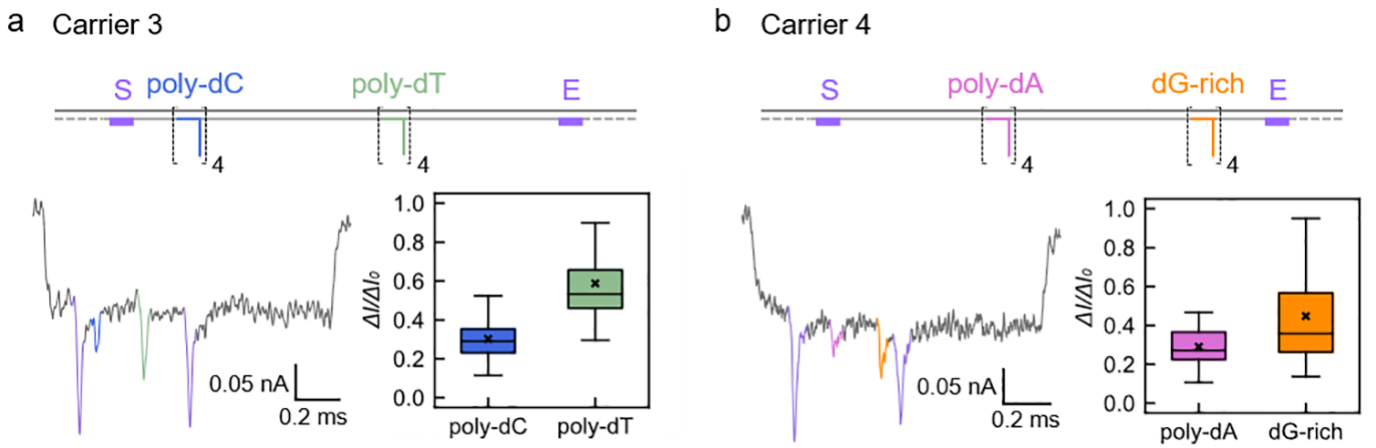
Studies on the dependence of the nanopore current signal on the overhang sequence. It is technically challenging to synthesize oligonucleotides of pure G, so repeats of “GGGGA” (dG-rich) were used here. Other DNA overhangs all consist of only one type of nucleotide. (a) Schematic and nanopore measurement results of Carrier 3 with 30 nt poly dC and poly dT overhangs at positions A and C, respectively. (b) Schematic and nanopore measurement results of Carrier 4 with 30 nt poly dA and dG-rich overhangs at positions B and D, respectively.

The sequence is another important property of DNA that could affect the nanopore signal. Based on our nanopore measurement results of the above two carrier designs, 30 nt overhangs with four repeats were used in order to get a good signal-to-noise ratio. Three carriers (Figure 3 and Figure S16a) were designed to include all the categories of nucleotides and make them interrelated. In order to exclude any binding of free oligonucleotides to the DNA carrier, Carrier 3 had polydeoxycytidine (poly dC) at position A and poly dT at position C, while Carrier 4 had polydeoxyadenosine (poly dA) at position B and dG-rich at position D. The carriers were detected in different nanopores, and the data was analyzed as above. From the results in Figure 3a, we find that poly dT produces greater current drops than poly dC. The dG-rich overhangs show a similar trend when compared with poly dA strands as shown in Figure 3b. One explanation is that poly dC and poly dA oligonucleotides have a tendency to self-stack into secondary structures,^33−36^ thus reducing the volume of solution they displace when they translocate with the carrier backbone through the sensitive region of the nanopore. A careful assessment of the data for dG-rich blockade signals reveals visibly larger variability (compare boxplots in Figure 3a,b and scatterplots in Figure S16c) of the current signals. The origin of the variability may be partly caused by interactions with neighboring overhangs. Stacked guanine bases are likely to interact with each other and form complicated 3D structures.^37−39^ A more detailed analysis can be found in Supporting Information S3.3 to help verify this explanation. We also demonstrate current signals of poly dT are larger than those of overhangs with a random sequence of the same length in Supporting Information S3.3.

## Dependence on the Molecular Weight, Volume, and Flexibility of DNA

**Figure 4:**
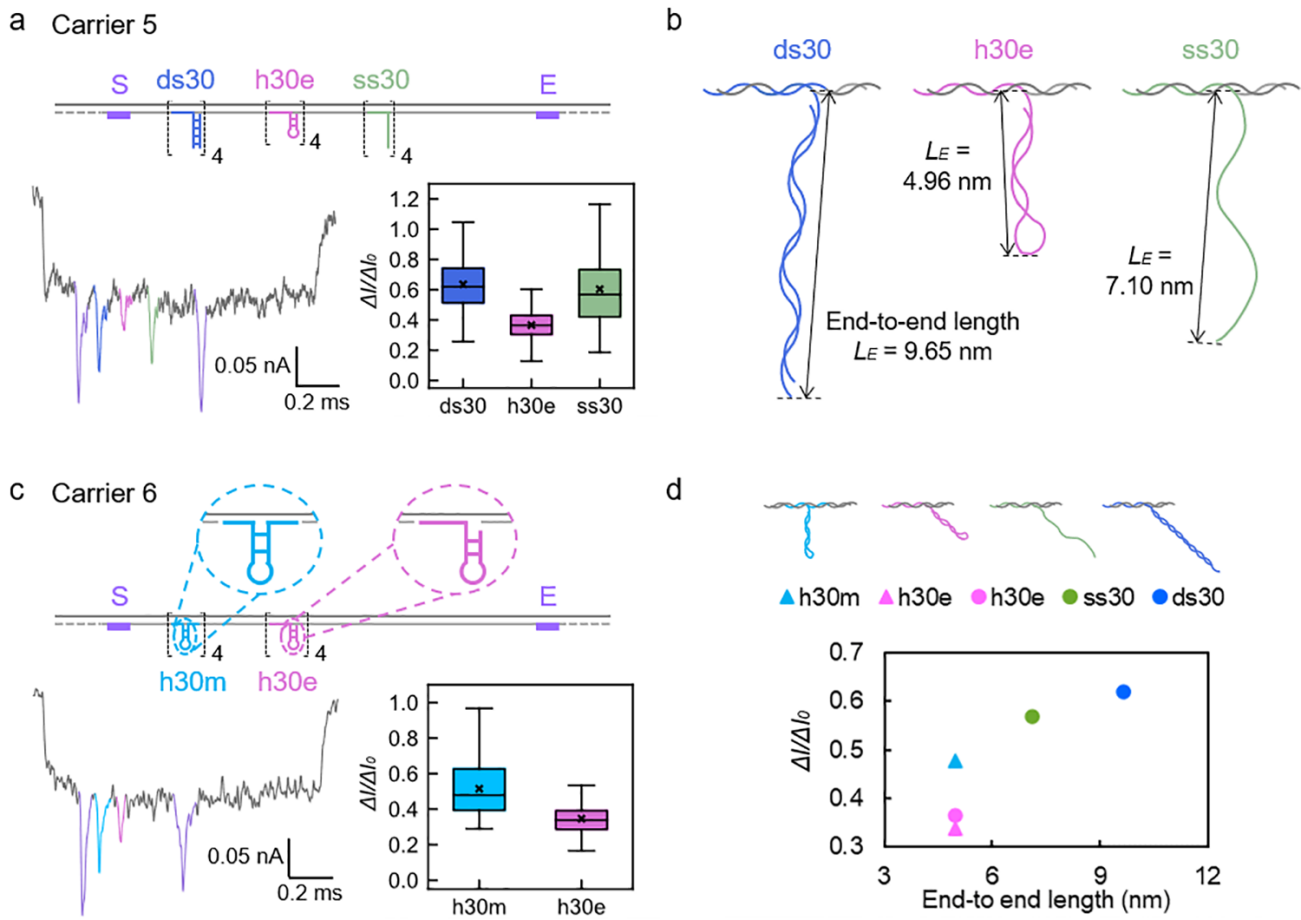
Studies on the dependence of nanopore current signal on molecular weight, volume, and flexibility of DNA structures. (a) Schematic and measurement results of Carrier 5 studying the dependence of nanopore current signals on DNA hybridization. “ds30” and “ss30” refer to dsDNA and ssDNA overhangs with a length of 30 bp or 30 nt. And “h30e” refers to the hairpin structure formed by self-folding of 30 nt ssDNA at one end of the staple strand. (b) Detailed structures and end-to-end lengths (*L*_E_) of the designed overhangs calculated from the worm-like chain (WLC) model. (c) Schematic and measurement result of Carrier 6 studying the dependence of nanopore current signals on the flexibility of DNA structure. “h30m” refers to the hairpin which has the same length as h30e but is designed in the middle of the staple strand and anchored to the carrier scaffold with both ends. (d) Relationship between *L*_E_ of DNA nanostructures and the Δ*I*/Δ*I*_0_ they generate. Different colored circles are from measurement results of Carrier 5. Blue and pink triangles are from measurement results of Carrier 6.

Following our studies on different ssDNA overhangs and their current drops, we further investigated a comparison of ssDNA to dsDNA. One major point to consider is whether molecular weight contributes to the intensity of nanopore signals and how it affects them. dsDNA has almost twice the molecular weight of ssDNA with the same number of bp/nt. Therefore, a comparison between dsDNA and ssDNA is ideal for investigating the effect that molecular weight has on nanopore signals. Another attraction of introducing the comparison between ssDNA and dsDNA is that the featured double-helix structure of dsDNA shortens its maximum length compared to fully extended ssDNA but, at the same time, provides additional stiffness. We decided to study the difference using nanopore measurements of dsDNA and ssDNA overhang structures located on the same Carrier 5 as shown in Figure 4a. The carrier contains a dsDNA (ds30) 30 bp in length at position A and a 30 nt poly dT (ss30) at position C. At position B, we designed a small hairpin (h30e) with a total length of 30 nt (13 bp for the stem and 4 nt for loop).

Surprisingly, the typical nanopore event in Figure 4a shows that the hairpin structure at position B, formed by self-folding of 30 nt ssDNA, results in the lowest peak depth in almost all of the events. Although the 30 nt ssDNA structure at position C generates greater current drops in some cases, it fails to give as strong nanopore signals as the 30 bp dsDNA structure at position A overall. As the 30 nt hairpin and poly dT structure have very similar molecular weights, we can conclude that molecular weight is only one aspect that explains signal generation. Results shown in Figure S18 prove that the above observation does not change with the variation in the diameter of the nanopore. To analyze the results quantitatively, and considering that DNA molecules can be described as semiflexible polymers, we decide to apply the worm-like chain (WLC)^40−42^ model in polymer physics to our system to account for the flexibility of DNA molecules (more details see Supporting Information S2.3). The end-to-end length (*L*_E_) calculated from the WLC model can be used to estimate the flexibility of DNA and the size of the average volume in the nanopore. For constructs of the same nominal volume, the one with a longer *L*_E_ further reduces the ion mobility in the proximity compared to the compact one with a shorter *L*_E_, leading to more significant nanopore current signals.^43−45^*L*_E_ values and sketches of the overhangs on Carrier 5 are shown in Figure 4b. Notably, there is still no generally recognized approach to calculate *L*_E_ for loop-shaped polymers, so the 4-dT loop at the end of the h30e hairpin is approximated by 2 bp dsDNA, that is to say the entire h30e structure is approximated by a 15 bp dsDNA. The *L*_E_ values show that the hairpin has the smallest signal as expected. However, a shorter *L*_E_ but comparable Δ*I*/Δ*I*_0_ of poly dT to dsDNA demonstrates that molecular weight cannot solely explain the signal depth, but the flexibility and volume of the DNA strands are important as well. Therefore, we designed another Carrier 6 as shown in Figure 4c to see if the mode of binding of the DNA structure to the carrier is important.

In Carrier 6, the h30m structure at position A contains a hairpin of the same length as the h30e structure at position B in Carrier 5 and Carrier 6, but it is designed in the middle of the staple strand and anchored to the scaffold with both ends. In contrast, h30e is formed by the self-folding of ssDNA overhangs at one end of the oligonucleotides, making them partly disconnected from the scaffold and offering them more freedom to spin around. As a result, the spatial morphology of h30m is relatively more fixed than that of h30e and is forced to “stand up” rather than “lie down”. Nanopore measurement results of Carrier 6 shown in Figure 4c suggest that the stiffer h30m structure in general brings about more significant current drops than the more pliable h30e structure, proving the importance of DNA flexibility in nanopore signal generation. The relationship between *L*_E_ of the above structures on Carrier 5 and Carrier 6 and their corresponding Δ*I*/Δ*I*_0_, as shown in Figure 4d, strengthens our result that the nanopore signal is determined by both volume and flexibility of DNA structures.

## Dependence on Unpaired Nucleotides in DNA Structures

**Figure 5:**
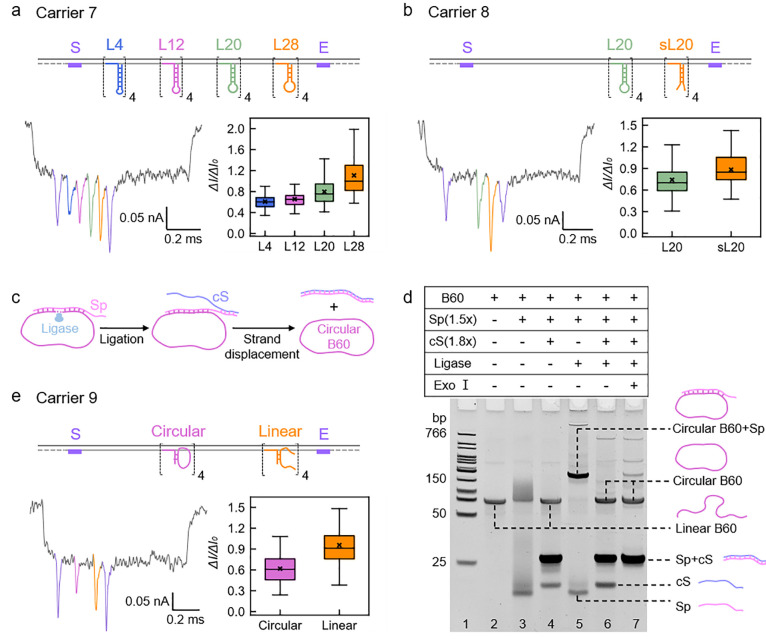
Studies on the dependence of nanopore current signal on unpaired nucleotides in DNA structures. (a) Schematic and measurement result of Carrier 7 with stem-loop structures of the same nucleotide numbers but different stem-loop ratios. “L4” to “L28” refer to structures consisting of “38 bp dsDNA + 4T loop”, “34 bp dsDNA + 12T loop”, “30 bp dsDNA + 20T loop”, and “26 bp dsDNA + 28T loop”, respectively. Loops are formed by self-folding of ssDNA with sequential T in the middle part. (b) Schematic and measurement result of Carrier 8 with a stem-loop and ssDNA splits. “sL20” refers to the L20-like structure whose 20 nt loop splits into two 10 nt poly dT strands. (c) Schematic of the synthesis process of circular ssDNA molecules. B60 is a 60 nt strand used to form circular ssDNA at position B on the carrier. Sp is the splint strand of B60. cS is the strand complementary to Sp that triggers the toehold-mediated strand displacement. (d) 15% native polyacrylamide gel electrophoresis (PAGE) gel result of circular B60. Lane 1 was added with a low molecular weight DNA ladder as a control. Lane 2 to lane 7 were added with different intermediate products of circular DNA preparation to demonstrate the successful synthesis of circular B60. (e) Schematic and measurement result of Carrier 9 discriminating between circular DNA and linear DNA.

The clear difference in nanopore signals of ssDNA and hairpin with the same nucleotide numbers inspired us to further explore the effect of unpaired bases in DNA structures on their current signals. The hairpin or stem-loop structure is a well-known secondary DNA structure and plays an essential role in biomolecular recognition and gene regulation.^46,47^ Following our results on dsDNA and ssDNA, it is easy to conclude that a DNA loop displaces a different volume in solution from dsDNA with the same number of nucleotides. When the number of unpaired nucleotides is limited, the difference in volume between a small loop and its corresponding dsDNA is not significant and thus can be neglected (e.g., the 4-dT loop in the h30e hairpin structure shown in Figure 4 can be reasonably approximated by 2 bp dsDNA). However, as the number of unpaired nucleotides increases, the difference becomes more significant as the ssDNA segment comprising a big loop is likely to move much more freely than paired dsDNA and accordingly occupies a considerably larger volume. As shown in Figure 5a, four hairpins with the same nucleotide numbers but different stem-loop ratios are designed on the same carrier for comparison. From the results, we can see that as the proportion of unpaired nucleotides making up the stem-loop structure increases, the consequent average/median peak depth keeps going up within the range of this work. The results confirm our prediction that unzipping the double strand to generate more unpaired bases can increase the molecular volume of DNA and contribute to the deeper current signals.

However, loops are obviously still not as extendable as single strands without terminal restrictions. To further study the flexibility of DNA structures on the nanopore signal, the 20 nt loop in the L20 structure at position C is split into two 10 nt single strands at position D on Carrier 8 (Figure 5b) and the two free ends give rise to even greater drops in the current trace, proving that loop-shaped DNA can gain a higher degree of freedom after splitting into halves.

## Discrimination of Circular DNA from Linear DNA

Circular ssDNA, as a class of ssDNA featured with covalently closed topology, have been widely applied in nucleic acid therapeutics and diagnostics as the backbone of virus genomes, aptamers, protein donors, nanostructure templates, microRNA (miRNA) inhibitors, and many more.^29,48−51^ However, there have been few trials on detecting these special DNA molecules by using nanopore sensors. The result in Figure 5b indicates that loop-shaped DNA generates different nanopore current signals from split ssDNA of the same number of nucleotides; hence we set out to show that we can distinguish circular and linear ssDNA with our nanopore sensor design. Considering the interest in detecting native circular DNA, our approach may greatly benefit medical research and disease treatment if we can identify the existence of circular DNA.

We prepared a circular ssDNA of 60 nucleotides from linear ssDNA (B60) using T4 ligase (Figure 5c). In the presence of the splint strand (Sp), the 5′ and 3′ ends of B60 were brought close by hybridization with Sp and covalently bound together by T4 ligase. A toehold end was reserved on Sp, so it could be easily removed from the DNA ring after ligation by adding a complementary strand (cS) to trigger the toehold-mediated strand displacement reaction.^52^ These reactions were investigated by 15% native polyacrylamide gel electrophoresis (PAGE) (Figure 5d). In lane 5, a new band with a low gel mobility appeared after the addition of T4 ligase. It should be circular B60 with Sp bound on it. By adding excess cS, Sp was removed from B60 and formed the dark band of the double strand in lane 6. Meanwhile, circular B60 shifted back to the same position as linear B60 in the gel, which indicated that the gel cannot separate these two structures. To further prove that circular DNA was formed, exonuclease I (Exo I) was added. In lane 7, the band of cS disappeared as expected, and bands of dsDNA Sp•cS and circular ssDNA B60 were left. The application of Exo I can also eliminate the unreacted linear ssDNA and guarantee the high purity of circular B60 we collected from the gel.

The circular ssDNA B60 and another linear ssDNA of the same length, which we call D60, were bound to Carrier 9 at position B and position D, respectively, for comparison (Figure 5e). We find that the current drops induced by these two structures can be easily discriminated at a statistical level because end-free linear DNA on average displaces a larger volume of solution than their circular counterparts when translocating through the nanopore. Not only that, we anticipate such nanopore signal differences between dsDNA, ssDNA, and loop DNA can be used to identify more complex DNA structures. Following our experiments, the detection of clinical circular DNA samples by nanopore sensors and simple hybridization to DNA carriers will be possible in the near future. Our results may also guide the development of models for predicting nanopore current signals including the volume, flexibility, and topology of the nucleic acid targets.

## Conclusion

In this work, we demonstrate clear differences in the blockade currents of DNA in the nanopore, depending on many factors that have not been systematically studied so far. Based on controlled translocation of carefully designed DNA nanostructures through glass nanopores, we show that the volume, topology, and flexibility of DNA overhangs are the decisive factors of nanopore signals, but not only their molecular weight. In addition, we can regulate these properties of the analyte DNA by changing its length, strand duplications, sequence, unpaired nucleotides, rigidity, etc. This finding offers valuable guidance for the development of new nanopore-based nucleic acid sensing and information storage systems. Our work also demonstrates the potential of the carrier-based nanopore sensing platform on studying DNA as well as RNA structures.^53^ In the medical field, differentiating between circular and linear DNA can allow this system to be translated to be used as an effective diagnostic tool.

## Supporting Information Available

Detailed sketches and recipes of carriers, supporting theories, supplementary experiments, experimental details (PDF)

## References

[ref1] Dekker C. Solid-state nanopores. Nat. Nanotechnol. 2007, 2, 209–215. 10.1038/nnano.2007.27. 18654264

[ref2] Nam S. W.; Rooks M. J.; Kim K. B.; Rossnagel S. M. Ionic field effect transistors with sub-10 nm multiple nanopores. Nano Lett. 2009, 9, 2044–2048. 10.1021/nl900309s. 19397298

[ref3] McNally B.; Singer A.; Yu Z.; Sun Y.; Weng Z.; Meller A. Optical recognition of converted DNA nucleotides for single-molecule DNA sequencing using nanopore arrays. Nano Lett. 2010, 10, 2237–2244. 10.1021/nl1012147. 20459065 PMC2883017

[ref4] Yamazaki H.; Hu R.; Henley R. Y.; Halman J.; Afonin K. A.; Yu D.; Zhao Q.; Wanunu M. Label-free single-molecule thermoscopy using a laser-heated nanopore. Nano Lett. 2017, 17, 7067–7074. 10.1021/acs.nanolett.7b03752. 28975798

[ref5] Fanget A.; Traversi F.; Khlybov S.; Granjon P.; Magrez A.; Forró L.; Radenovic A. Nanopore integrated nanogaps for DNA detection. Nano Lett. 2014, 14, 244–249. 10.1021/nl403849g. 24308689

[ref6] Liu S.; Xie B.; Zhong C.; Wang J.; Ying Y.; Long Y. An advanced optical–electrochemical nanopore measurement system for single-molecule analysis. Rev. Sci. Instrum. 2021, 92, 121301 10.1063/5.0067185. 34972456

[ref7] Merchant C. A.; Healy K.; Wanunu M.; Ray V.; Peterman N.; Bartel J.; Fischbein M. D.; Venta K.; Luo Z.; Johnson A. C.; Drndić M. DNA translocation through graphene nanopores. Nano Lett. 2010, 10, 2915–2921. 10.1021/nl101046t. 20698604

[ref8] Wanunu M.; Dadosh T.; Ray V.; Jin J.; McReynolds L.; Drndic M. Rapid electronic detection of probe-specific microRNAs using thin nanopore sensors. Nat. Nanotechnol. 2010, 5, 807–814. 10.1038/nnano.2010.202. 20972437

[ref9] Kim M. J.; Wanunu M.; Bell D. C.; Meller A. Rapid fabrication of uniformly sized nanopores and nanopore arrays for parallel DNA analysis. Adv. Mater. 2006, 18, 3149–3153. 10.1002/adma.200601191.

[ref10] Kwok H.; Briggs K.; Tabard-Cossa V. Nanopore fabrication by controlled dielectric breakdown. PLoS One 2014, 9, e92880 10.1371/journal.pone.0092880. 24658537 PMC3962464

[ref11] Feng J.; Liu K.; Graf M.; Lihter M.; Bulushev R. D.; Dumcenco D.; Alexander D. T. L.; Krasnozhon D.; Vuletic T.; Kis A.; Radenovic A. Electrochemical reaction in single layer MoS_2_: nanopores opened atom by atom. Nano Lett. 2015, 15, 3431–3438. 10.1021/acs.nanolett.5b00768. 25928894

[ref12] Liu K.; Pan C.; Kuhn A.; Nievergelt A. P.; Fantner G. E.; Milenkovic O.; Radenovic A. Detecting topological variations of DNA at single-molecule level. Nat. Commun. 2019, 10, 3 10.1038/s41467-018-07924-1. 30602774 PMC6315031

[ref13] Gilboa T.; Zrehen A.; Girsault A.; Meller A. Optically-monitored nanopore fabrication using a focused laser beam. Sci. Rep. 2018, 8, 1–10. 10.1038/s41598-018-28136-z. 29950607 PMC6021433

[ref14] Pud S.; Verschueren D.; Vukovic N.; Plesa C.; Jonsson M. P.; Dekker C. Self-aligned plasmonic nanopores by optically controlled dielectric breakdown. Nano Lett. 2015, 15, 7112–7117. 10.1021/acs.nanolett.5b03239. 26333767 PMC4859154

[ref15] Xie Z.; Liu S.; Zhai Y. Study on the self-assembly and signal amplification ability of nucleic acid nanostructure with the nanopipette. J. Electroanal. Chem. 2022, 914, 116307 10.1016/j.jelechem.2022.116307.

[ref16] Wang D.; Xu X.; Zhou Y.; Li H.; Qi G.; Hu P.; Jin Y. Short-chain oligonucleotide detection by glass nanopore using targeting-induced DNA tetrahedron deformation as signal amplifier. Anal. Chim. Acta 2019, 1063, 57–63. 10.1016/j.aca.2019.02.058. 30967186

[ref17] Zhu Z.; Wu R.; Li B. Exploration of solid-state nanopores in characterizing reaction mixtures generated from a catalytic DNA assembly circuit. Chem. Sci. 2019, 10, 1953–1961. 10.1039/C8SC04875D. 30881624 PMC6385554

[ref18] Zhou Y.; Wu R.; Wang D.; Hu P.; Jin Y. Single-molecule translocation conformational sensing of multiarm DNA concatemers using glass capillary nanopore. ACS Sens 2019, 4, 3119–3123. 10.1021/acssensors.9b01880. 31797666

[ref19] Raveendran M.; Lee A. J.; Sharma R.; Wälti C.; Actis P. Rational design of DNA nanostructures for single molecule biosensing. Nat. Commun. 2020, 11, 4384 10.1038/s41467-020-18132-1. 32873796 PMC7463249

[ref20] Confederat S.; Sandei I.; Mohanan G.; Wälti C.; Actis P. Nanopore fingerprinting of supramolecular DNA nanostructures. Biophys. J. 2022, 121, 4882–4891. 10.1016/j.bpj.2022.08.020. 35986518 PMC9808562

[ref21] Chen K.; Jou I.; Ermann N.; Muthukumar M.; Keyser U. F.; Bell N. A. W. Dynamics of driven polymer transport through a nanopore. Nat. Phys. 2021, 17, 1043–1049. 10.1038/s41567-021-01268-2.

[ref22] Bell N. A. W.; Keyser U. F. Digitally encoded DNA nanostructures for multiplexed, single-molecule protein sensing with nanopores. Nat. Nanotechnol. 2016, 11, 645–651. 10.1038/nnano.2016.50. 27043197

[ref23] Zhu J.; Ermann N.; Chen K.; Keyser U. F. Image encoding using multi-level DNA barcodes with nanopore readout. Small 2021, 17, e2100711 10.1002/smll.202100711. 34133074

[ref24] Boskovic F.; Zhu J.; Chen K.; Keyser U. F. Monitoring G-quadruplex formation with DNA carriers and solid-state nanopores. Nano Lett. 2019, 19, 7996–8001. 10.1021/acs.nanolett.9b03184. 31577148

[ref25] Zhu J.; Bošković F.; Keyser U. F. Split G-quadruplexes enhance nanopore signals for simultaneous Identification of multiple nucleic acids. Nano Lett. 2022, 22, 4993–4998. 10.1021/acs.nanolett.2c01764. 35730196 PMC9228402

[ref26] Chen K.; Kong J.; Zhu J.; Ermann N.; Predki P.; Keyser U. F. Digital data storage using DNA nanostructures and solid-state nanopores. Nano Lett. 2019, 19, 1210–1215. 10.1021/acs.nanolett.8b04715. 30585490

[ref27] Helinski D. R.; Clewell D. Circular DNA. Annu. Rev. Biochem. 1971, 40, 899–942. 10.1146/annurev.bi.40.070171.004343. 4942224

[ref28] Li R.; Wang Y.; Li J.; Zhou X. Extrachromosomal circular DNA (eccDNA): an emerging star in cancer. Biomark. Res. 2022, 10, 53 10.1186/s40364-022-00399-9. 35883211 PMC9327165

[ref29] Hino S. TTV, a new human virus with single stranded circular DNA genome. Rev. Mol. Virol. 2002, 12, 151–158. 10.1002/rmv.351. 11987140

[ref30] Kim S. H.; Lee T. H. Conformational dynamics of poly (T) single-stranded DNA at the single-molecule Level. J. Phys. Chem. Lett. 2021, 12, 4576–4584. 10.1021/acs.jpclett.1c00962. 33970634 PMC10034556

[ref31] Liang X.; Kuhn H.; Frank-Kamenetskii M. D. Monitoring single-stranded DNA secondary structure formation by determining the topological state of DNA catenanes. Biophys. J. 2006, 90, 2877–2889. 10.1529/biophysj.105.074104. 16461397 PMC1414558

[ref32] Maffeo C.; Ngo T. T.; Ha T.; Aksimentiev A. A coarse-grained model of unstructured single-stranded DNA derived from atomistic simulation and single-molecule experiment. J. Chem. Theory Comput. 2014, 10, 2891–2896. 10.1021/ct500193u. 25136266 PMC4132850

[ref33] Roth E.; Glick Azaria A.; Girshevitz O.; Bitler A.; Garini Y. Measuring the conformation and persistence length of single-stranded DNA using a DNA origami structure. Nano Lett. 2018, 18, 6703–6709. 10.1021/acs.nanolett.8b02093. 30352164

[ref34] Goddard N. L.; Bonnet G.; Krichevsky O.; Libchaber A. Sequence dependent rigidity of single stranded DNA. Phys. Rev. Lett. 2000, 85, 2400 10.1103/PhysRevLett.85.2400. 10978020

[ref35] Mills J. B.; Vacano E.; Hagerman P. Flexibility of single-stranded DNA: use of gapped duplex helices to determine the persistence lengths of poly (dT) and poly (dA). J. Mol. Biol. 1999, 285, 245–257. 10.1006/jmbi.1998.2287. 9878403

[ref36] Rizzuto F. J.; Dore M. D.; Rafique M. G.; Luo X.; Sleiman H. F. DNA sequence and length dictate the assembly of nucleic acid block copolymers. J. Am. Chem. Soc. 2022, 144, 12272–12279. 10.1021/jacs.2c03506. 35762655

[ref37] Lech C. J.; Heddi B.; Phan A. T. Guanine base stacking in G-quadruplex nucleic acids. Nucleic Acids Res. 2013, 41, 2034–2046. 10.1093/nar/gks1110. 23268444 PMC3561957

[ref38] Lipps H. J.; Rhodes D. G-quadruplex structures: in vivo evidence and function. Trends Cell Biol. 2009, 19, 414–422. 10.1016/j.tcb.2009.05.002. 19589679

[ref39] Biffi G.; Tannahill D.; McCafferty J.; Balasubramanian S. Quantitative visualization of DNA G-quadruplex structures in human cells. Nat. Chem. 2013, 5, 182–186. 10.1038/nchem.1548. 23422559 PMC3622242

[ref40] Muthukumar M. Polymer Translocation; CRC press, 2016.

[ref41] Doi M.; Edwards S. F.; Edwards S. F. The Theory of Polymer Dynamics; Oxford University Press, 1988; Vol. 73.

[ref42] Rubinstein M.; Colby R. H. Polymer Physics; Oxford University Press, 2003; Vol. 23.

[ref43] Wilson J.; Sarthak K.; Si W.; Gao L.; Aksimentiev A. Rapid and accurate determination of nanopore ionic current using a steric exclusion model. Acs Sens 2019, 4, 634–644. 10.1021/acssensors.8b01375. 30821441 PMC6489136

[ref44] Si W.; Aksimentiev A. Nanopore sensing of protein folding. ACS Nano 2017, 11, 7091–7100. 10.1021/acsnano.7b02718. 28693322 PMC5564329

[ref45] Kesselheim S.; Müller W.; Holm C. Origin of current blockades in nanopore translocation experiments. Phys. Rev. Lett. 2014, 112, 018101 10.1103/PhysRevLett.112.018101. 24483933

[ref46] Broude N. E. Stem-loop oligonucleotides: a robust tool for molecular biology and biotechnology. Trends Biotechnol 2002, 20, 249–256. 10.1016/S0167-7799(02)01942-X. 12007493

[ref47] Chen C.; Ridzon D. A.; Broomer A. J.; Zhou Z.; Lee D. H.; Nguyen J. T.; Barbisin M.; Xu N. L.; Mahuvakar V. R.; Andersen M. R.; Lao K. Q.; Livak K. J.; Guegler K. J. Real-time quantification of microRNAs by stem–loop RT–PCR. Nucleic Acids Res. 2005, 33, e179 10.1093/nar/gni178. 16314309 PMC1292995

[ref48] Jiang Y.; Pan X.; Chang J.; Niu W.; Hou W.; Kuai H.; Zhao Z.; Liu J.; Wang M.; Tan W. Supramolecularly engineered circular bivalent aptamer for enhanced functional protein delivery. J. Am. Chem. Soc. 2018, 140, 6780–6784. 10.1021/jacs.8b03442. 29772170 PMC6442730

[ref49] Iyer S.; Mir A.; Vega-Badillo J.; Roscoe B. P.; Ibraheim R.; Zhu L. J.; Lee J.; Liu P.; Luk K.; Mintzer E.; Guo D.; Brito J. S.; Emerson C. P.; Zamore P. D.; Sontheimer E. J.; Wolfe S. A. Efficient homology-directed repair with circular single-stranded DNA donors. CRISPR J. 2022, 5, 685–701. 10.1089/crispr.2022.0058. 36070530 PMC9595650

[ref50] Zhang L.; Zhu G.; Mei L.; Wu C.; Qiu L.; Cui C.; Liu Y.; Teng I. T.; Tan W. Self-assembled DNA immunonanoflowers as multivalent CpG nanoagents. ACS Appl. Mater. Interfaces 2015, 7, 24069–24074. 10.1021/acsami.5b06987. 26440045 PMC4898273

[ref51] Meng J.; Chen S.; Han J.; Tan Q.; Wang X.; Wang H.; Zhong W.; Qin Y.; Qiao K.; Zhang C.; Gao W.; Lei Y.; Liu H.; Liu Y.; Zhou H.; Sun T.; Yang C. Derepression of co-silenced tumor suppressor genes by nanoparticle-loaded circular ssDNA reduces tumor malignancy. Sci. Transl. Med. 2018, 10, eaao6321 10.1126/scitranslmed.aao6321. 29794062

[ref52] Srinivas N.; Ouldridge T. E.; Šulc P.; Schaeffer J. M.; Yurke B.; Louis A. A.; Doye J. P. K.; Winfree E. On the biophysics and kinetics of toehold-mediated DNA strand displacement. Nucleic Acids Res. 2013, 41, 10641–10658. 10.1093/nar/gkt801. 24019238 PMC3905871

[ref53] Bošković F.; Keyser U. F. Nanopore microscope identifies RNA isoforms with structural colours. Nat. Chem. 2022, 14, 1258–1264. 10.1038/s41557-022-01037-5. 36123450

