# Supplementary material for: DNA Volume, Topology, and Flexibility Dictate Nanopore Current Signals
Source: Nano Lett. 2023 Jul 24;23(15):7054–61. doi: 10.1021/acs.nanolett.3c01823 (PMC10416563; doi:10.1021/acs.nanolett.3c01823)
Supplement: Supplementary file 1 — nl3c01823_si_001.pdf [file nl3c01823_si_001.pdf]

Supporting Information

DNA volume, topology, and flexibility dictate nanopore current signals.

Yunxuan Li [a], Sarah E. Sandler [a], Ulrich F. Keyser [a]*, and Jinbo Zhu [a,b]*

[a] Cavendish Laboratory, University of Cambridge, JJ Thompson Avenue, Cambridge CB3 0HE, UK.
[b] School of Biomedical Engineering, Faculty of Medicine, Dalian University of Technology, No. 2 Linggong Road, Dalian 116024, China

E-mail: ufk20@cam.ac.uk; jinbozhu@dlut.edu.cn

S1 Details of DNA carriers

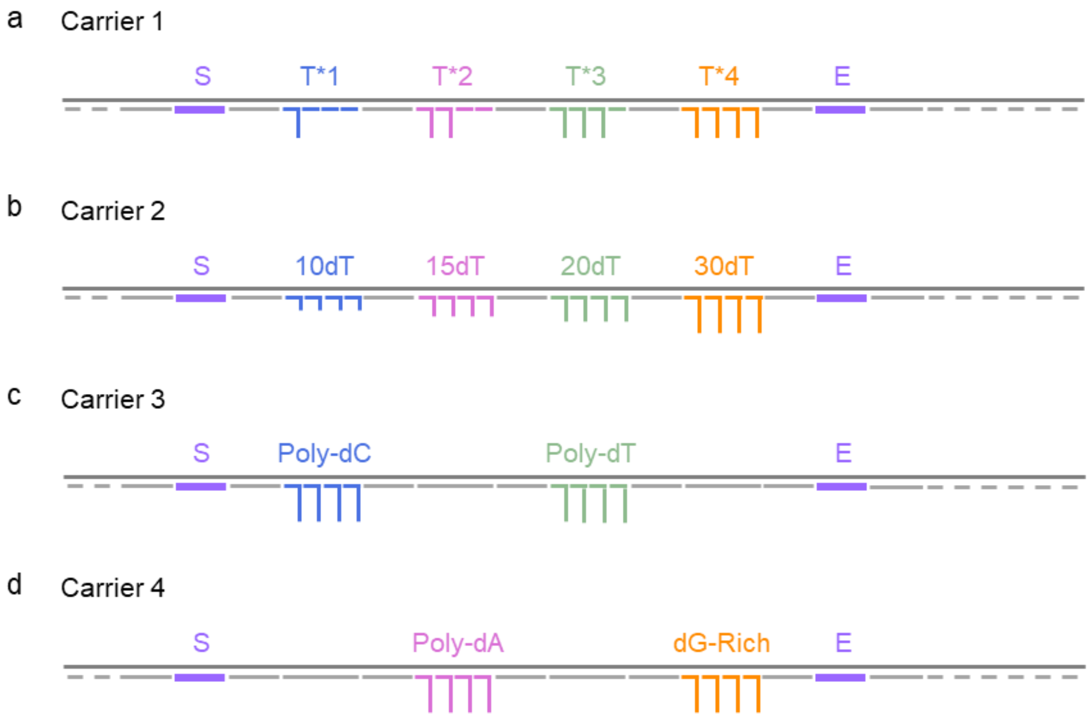

Figure S1 Detailed sketches of Carriers 1 to 4.

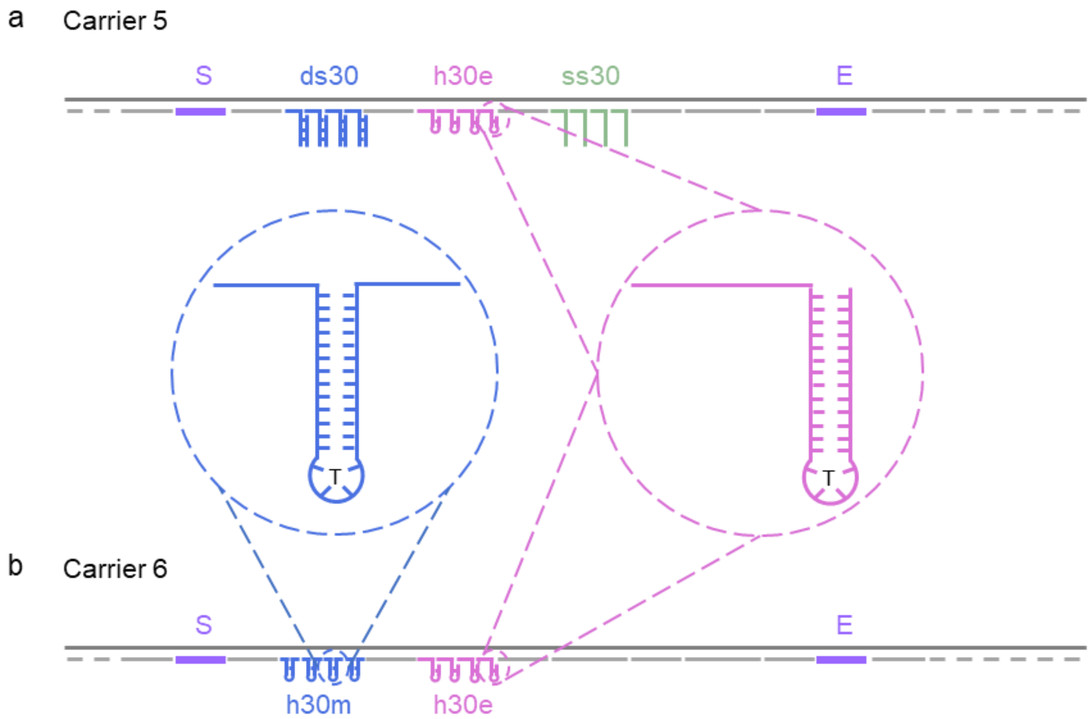

Figure S2 Detailed sketches of Carrier 5 and Carrier 6.

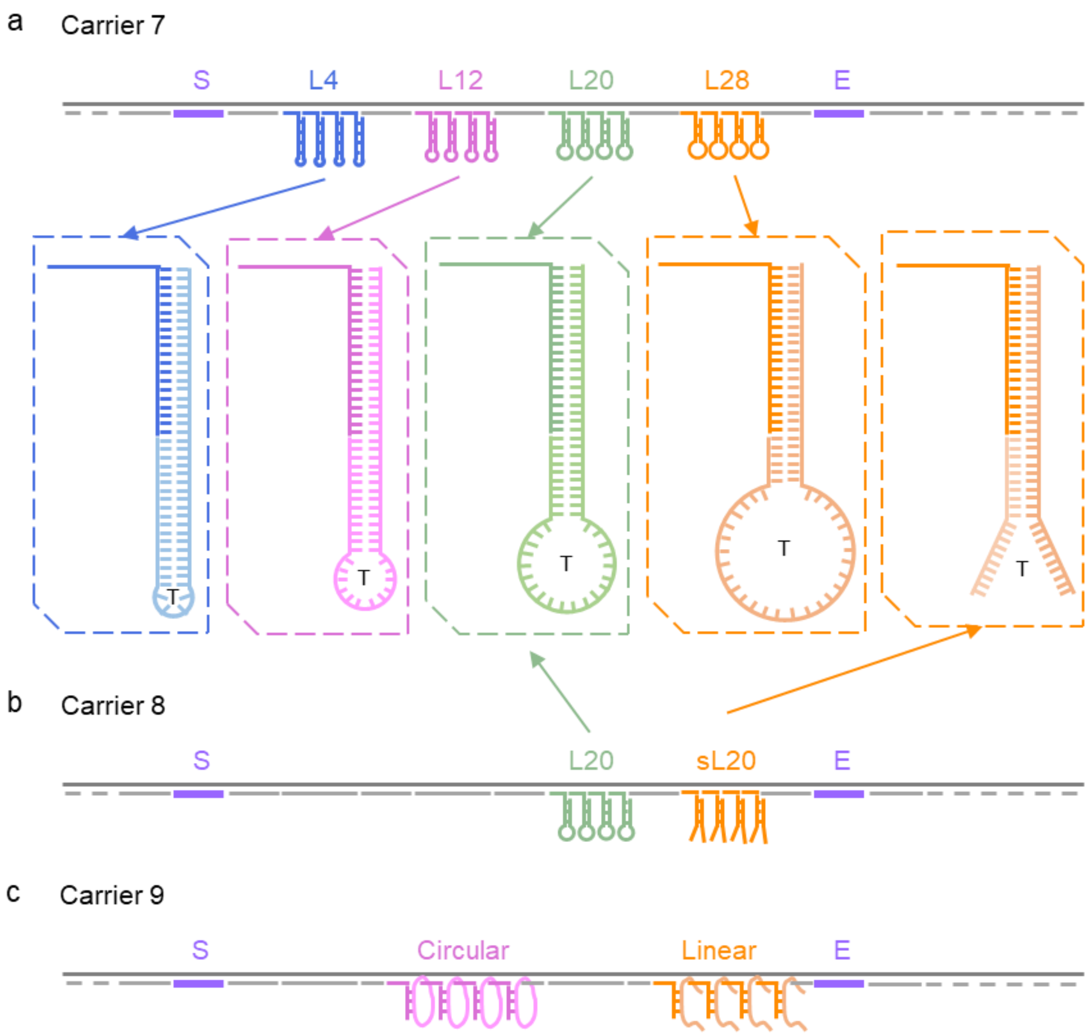

Figure S3 Detailed sketches of Carriers 7 to 9.

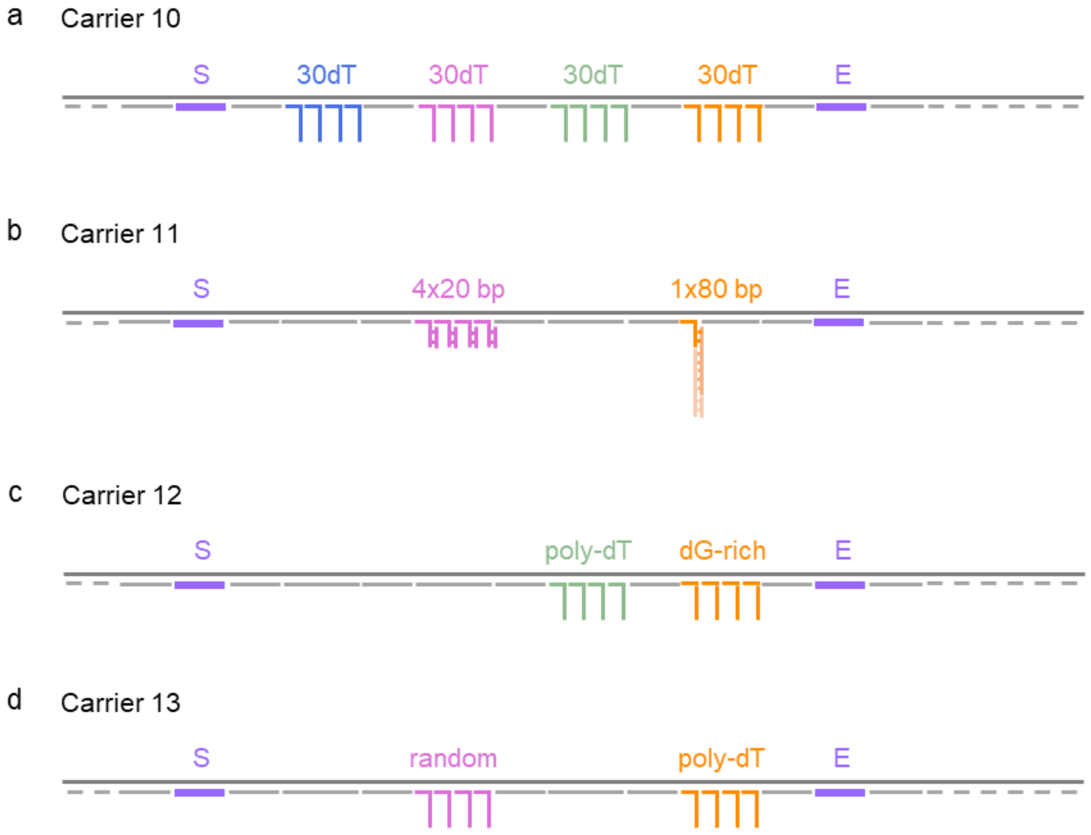

Figure S4 Detailed sketches of Carriers 10 to 13.

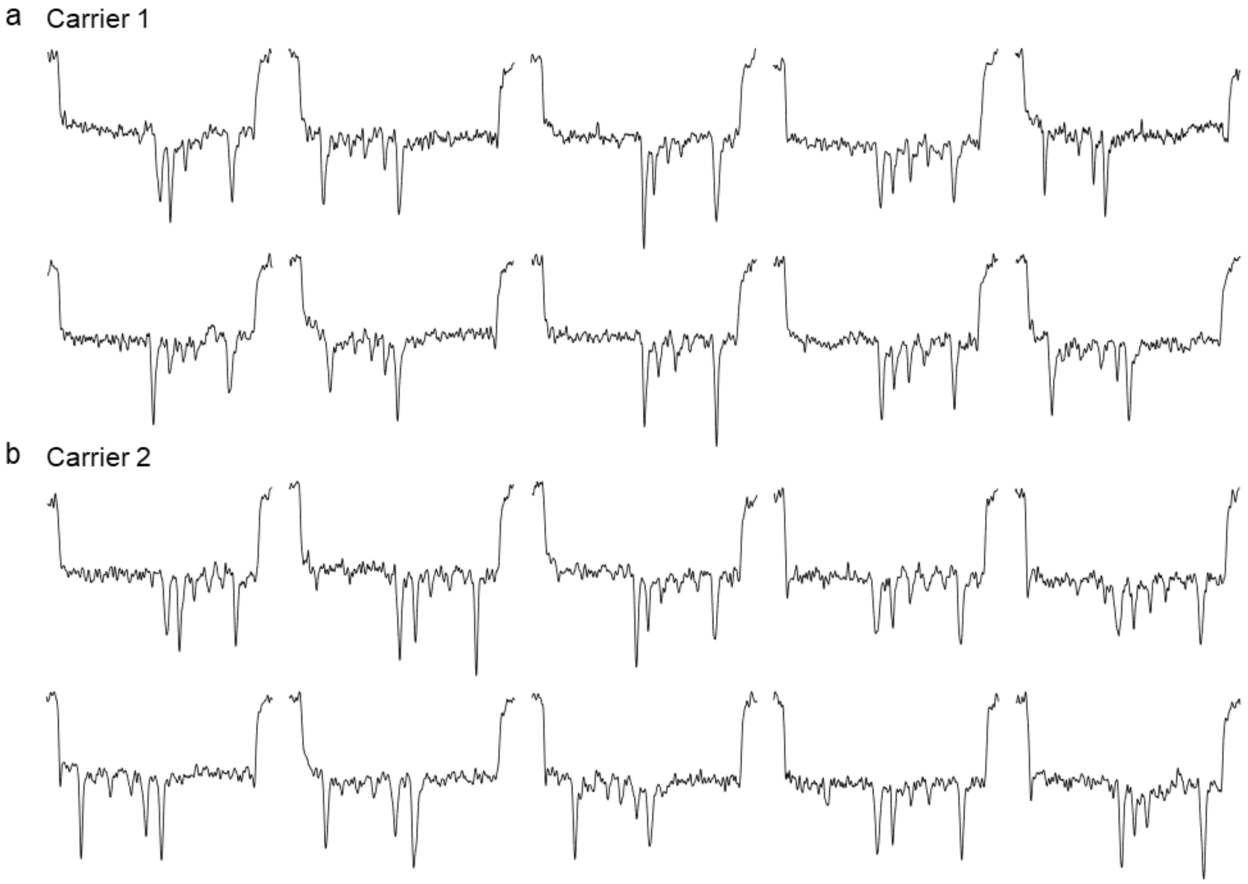

Figure S5 The first 10 unfolded translocation events of Carrier 1 and Carrier 2.

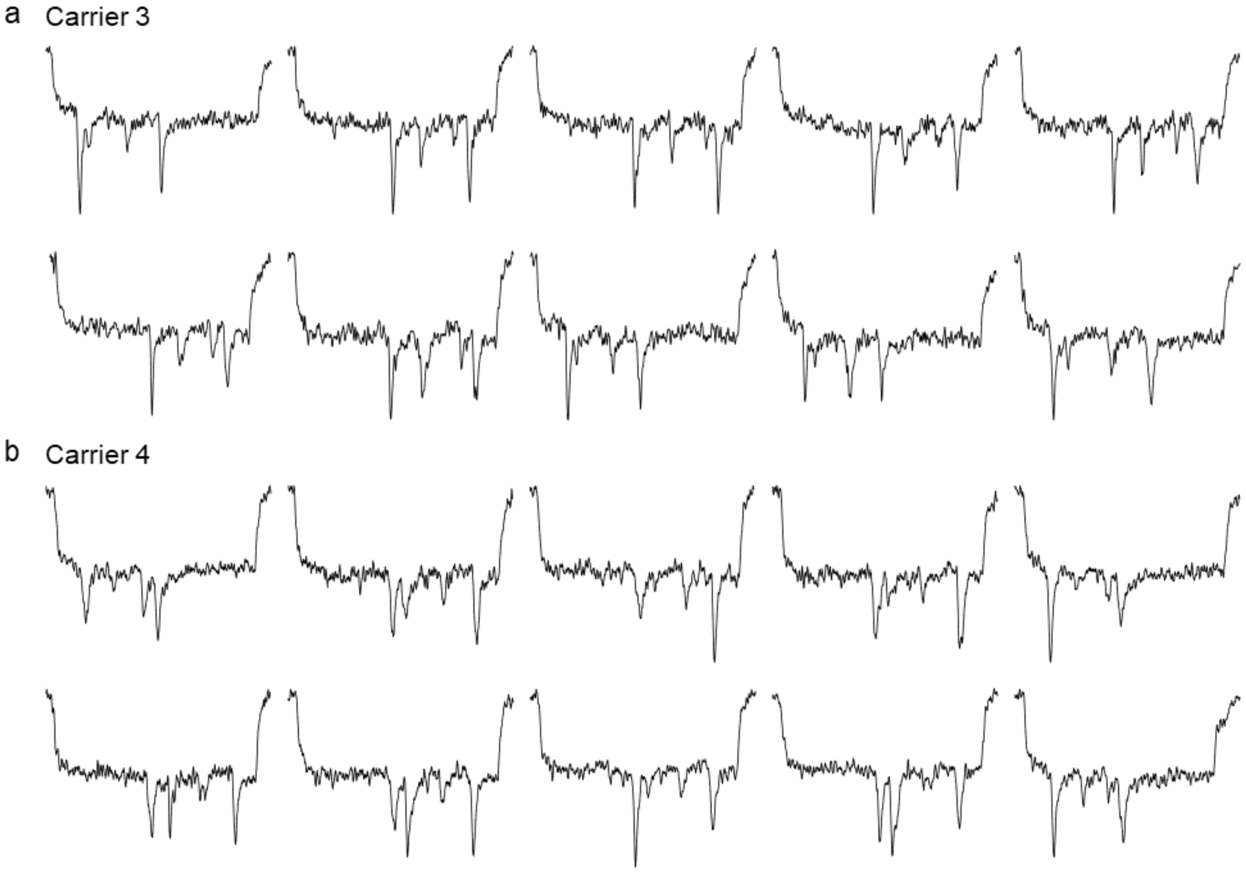

Figure S6 The first 10 unfolded translocation events of Carrier 3 and Carrier 4.

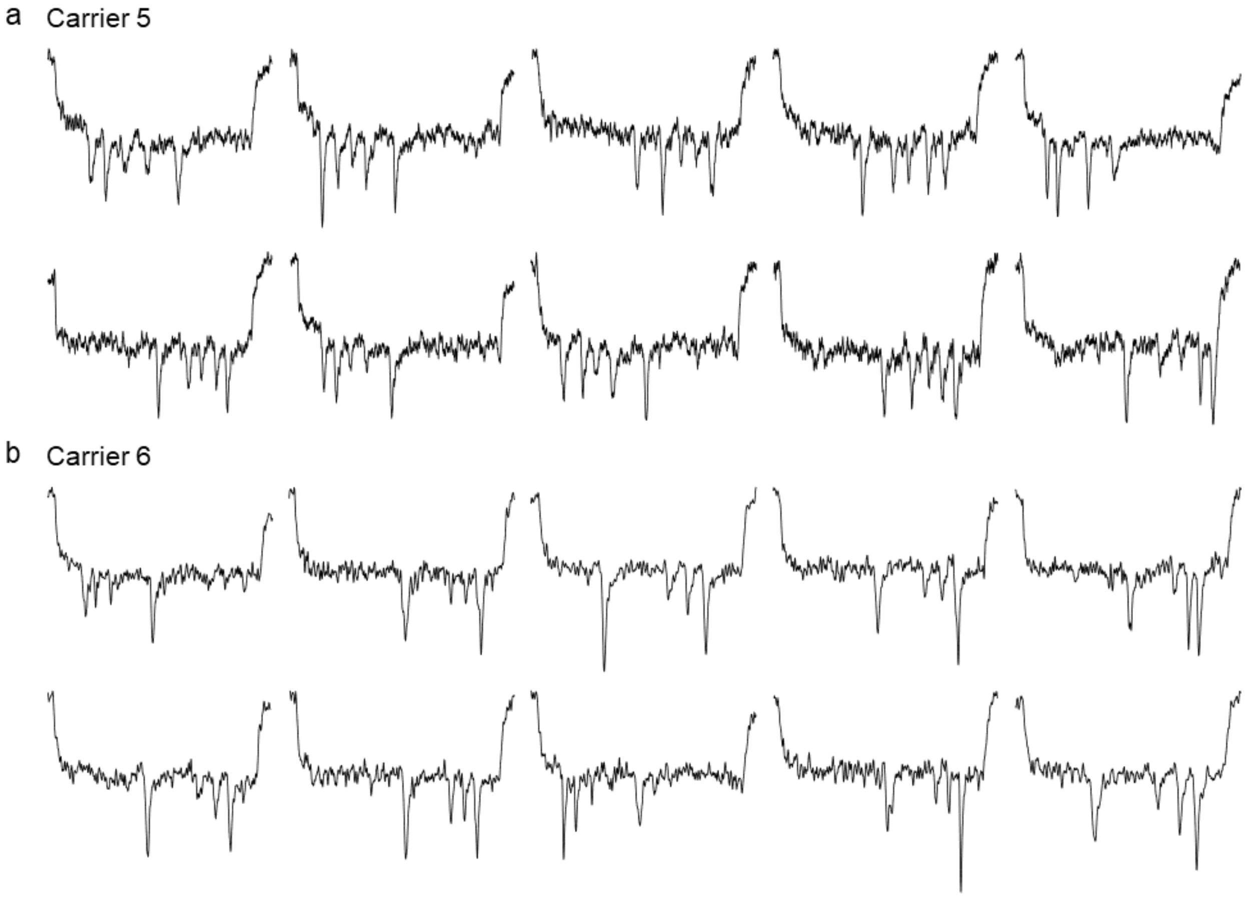

Figure S7 The first 10 unfolded translocation events of Carrier 5 and Carrier 6.

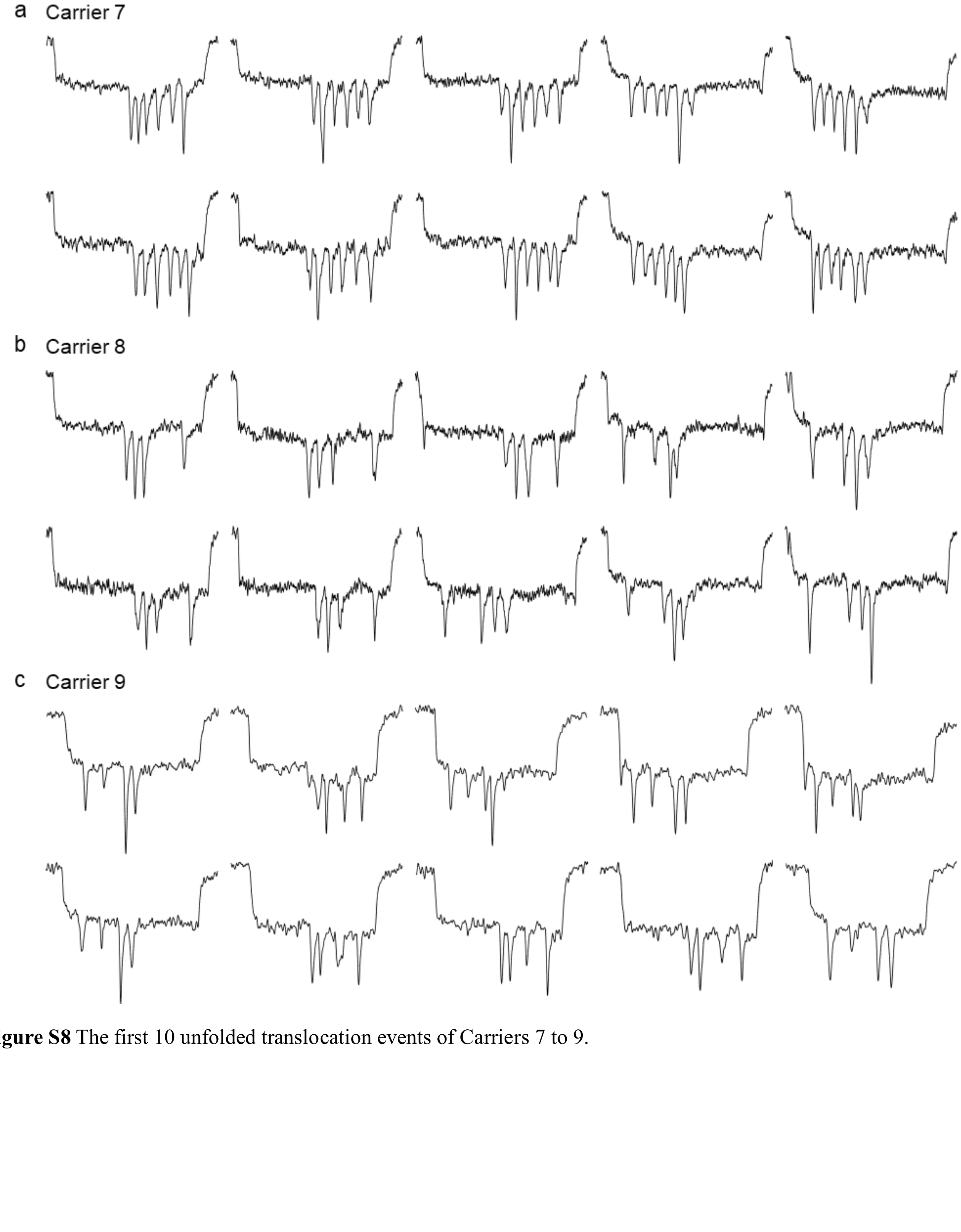

Figure S8 The first 10 unfolded translocation events of Carriers 7 to 9.

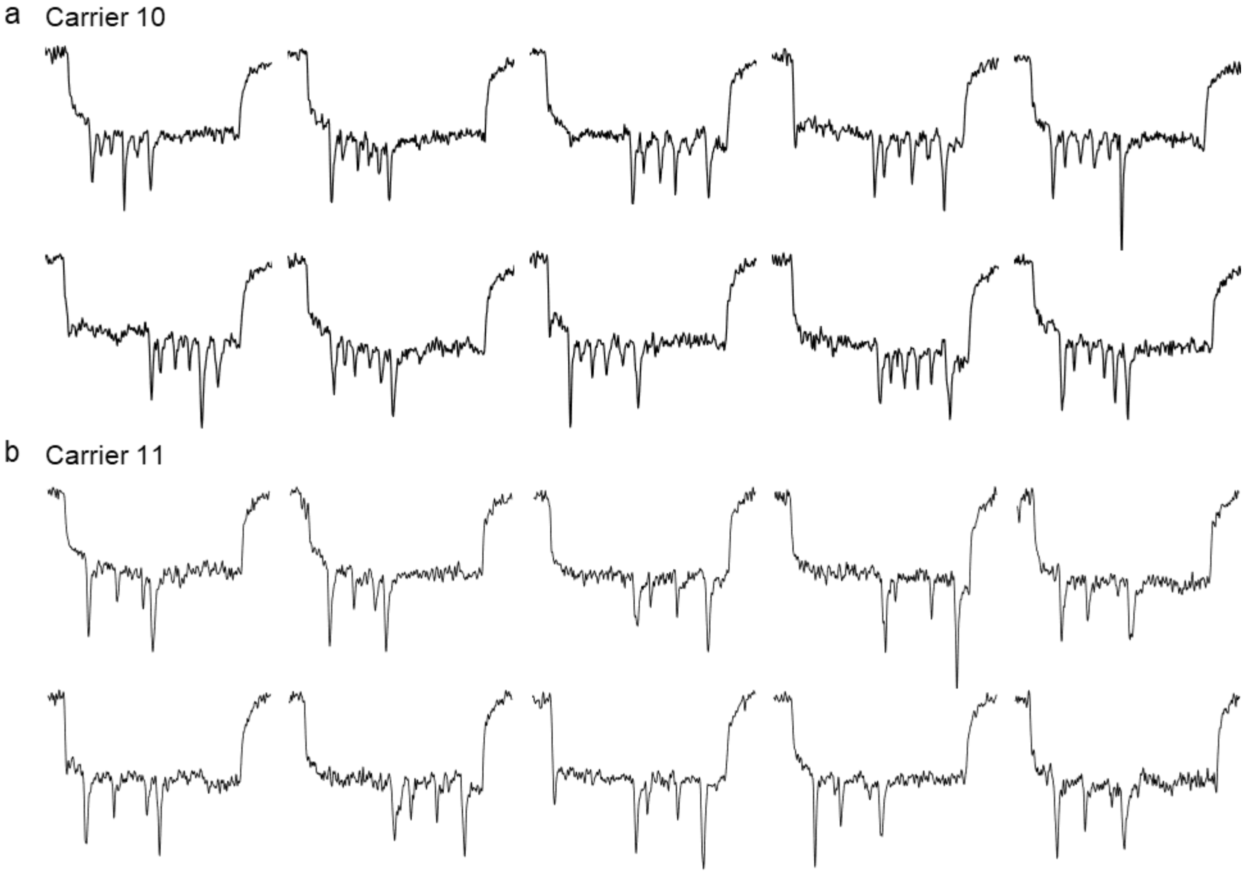

Figure S9 The first 10 unfolded translocation events of Carrier 10 and Carrier 11.

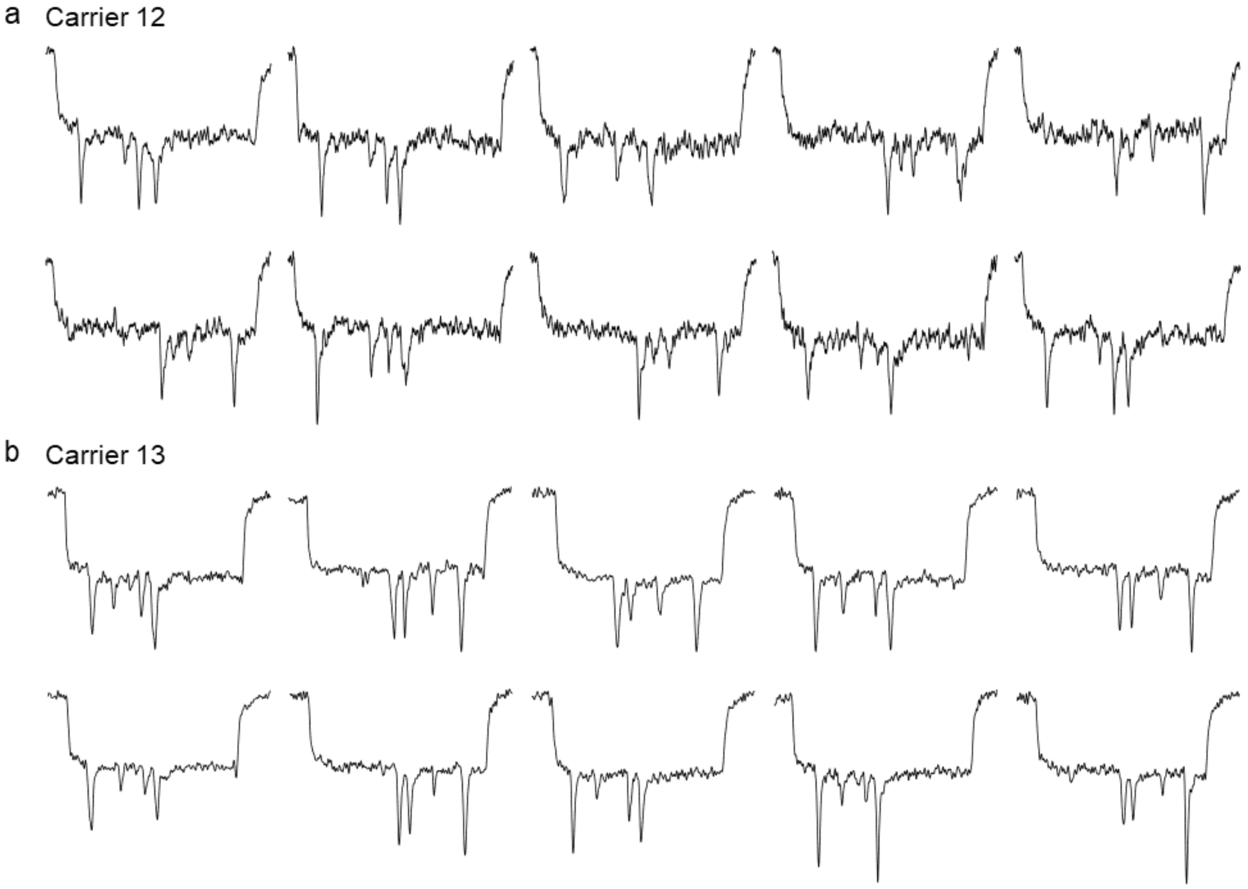

Figure S10 The first 10 unfolded translocation events of Carrier 12 and Carrier 13.

Table S1 Detailed recipes for synthesizing Carriers 1 to 13.

	Customized strands			
	Position A	Position B	Position C	Position D
Carrier 1	A1T30<br>AO2<br>AO3<br>AO4	B1T30<br>B2T30<br>BO3<br>BO4	C1T30<br>C2T30<br>C3T30<br>CO4	D1T30<br>D2T30<br>D3T30<br>D4T30
Carrier 2	A1T10<br>A2T10<br>A3T10<br>A4T10	B1T15<br>B2T15<br>B3T15<br>B4T15	C1T20<br>C2T20<br>C3T20<br>C4T20	D1T30<br>D2T30<br>D3T30<br>D4T30
Carrier 3	A1C30<br>A2C30<br>A3C30<br>A4C30		C1T30<br>C2T30<br>C3T30<br>C4T30	
Carrier 4		B1A30<br>B2A30<br>B3A30<br>B4A30		D1G30<br>D2G30<br>D3G30<br>D4G30
Carrier 5	AD1<br>AD2<br>AD3<br>AD4<br>+ cAD	BH1<br>BH2<br>BH3<br>BH4	C1T30<br>C2T30<br>C3T30<br>C4T30	
Carrier 6	AH1<br>AH2<br>AH3<br>AH4	BH1<br>BH2<br>BH3<br>BH4		
Carrier 7	AW1<br>AW2<br>AW3<br>AW4<br>+ HA4	BW1<br>BW2<br>BW3<br>BW4<br>+ HB12	CW1<br>CW2<br>CW3<br>CW4<br>+ HC20	DW1<br>DW2<br>DW3<br>DW4<br>+ HD28
Carrier 8			CW1<br>CW2<br>CW3<br>CW4<br>+ HC20	DW1<br>DW2<br>DW3<br>DW4<br>+ DWT10a<br>+ DWT10b
Carrier 9		BW1<br>BW2<br>BW3<br>BW4<br>+ circular B60		DW1<br>DW2<br>DW3<br>DW4<br>+ D60
Carrier 10	A1T30<br>A2T30<br>A3T30<br>A4T30	B1T30<br>B2T30<br>B3T30<br>B4T30	C1T30<br>C2T30<br>C3T30<br>C4T30	D1T30<br>D2T30<br>D3T30<br>D4T30
Carrier 11		BW1<br>BW2<br>BW3<br>BW4<br>+ B20		DW1<br>DO2<br>DO3<br>DO4<br>+ DE1, DE2, DE3
Carrier 12			C1T30<br>C2T30<br>C3T30<br>C4T30	D1G30<br>D2G30<br>D3G30<br>D4G30
Carrier 13		BR1<br>BR2<br>BR3<br>BR4		D1T30<br>D2T30<br>D3T30<br>D4T30

Table S2 Dumbbell oligonucleotides to replace staples 26-32 at reference S. The dumbbell motifs not bound to the carrier scaffold are marked in red.

1	ACATCACTTGTCCTCTTTTGAGGAACAAGTTTTCTTGTCCTGAGTAGA
2	AGAACTCAAATCCTCTTTTGAGGAACAAGTTTTCTTGTCTATCGGCCT
3	TGCTGGTAATTCCTCTTTTGAGGAACAAGTTTTCTTGTATCCAGAACA
4	ATATTACCGCTCCTCTTTTGAGGAACAAGTTTTCTTGTCAGCCATTGC
5	AACAGGAAAATCCTCTTTTGAGGAACAAGTTTTCTTGTACGCTCATGG
6	AAATACCTACTCCTCTTTTGAGGAACAAGTTTTCTTGTATTTTGACGC
7	TCAATCGTCTTCCTCTTTTGAGGAACAAGTTTTCTTGTGAAATGGATT
8	ATTTACATTGTCCTCTTTTGAGGAACAAGTTTTCTTGTGCAGATTCAC
9	CAGTCACACGTCCTCTTTTGAGGAACAAGTTTTCTTGTACCAGTAATA
10	AAAGGGACATTCCTCTTTTGAGGAACAAGTTTTCTTGTTCTGGCCAAC
11	AGAGATAGAATCCTCTTTTGAGGAACAAGTTTTCTTGTCCCTTCTGAC
12	CTGAAAGCGTAAGAATACGTGGCACAGACAATATTTTGAATGGCT

Table S3 Dumbbell oligonucleotides to replace staples 96-102 at reference E.

1	CTTGAGCCATTCCTCTTTTGAGGAACAAGTTTTCTTGTTTGGGAATTA
2	GAGCCAGCAATCCTCTTTTGAGGAACAAGTTTTCTTGTAATCACCAGT
3	AGCACCATTATCCTCTTTTGAGGAACAAGTTTTCTTGTCCATTAGCAA
4	GGCCGGAAACTCCTCTTTTGAGGAACAAGTTTTCTTGTGTCACCAATG
5	AAACCATCGATCCTCTTTTGAGGAACAAGTTTTCTTGTTAGCAGCACC
6	GTAATCAGTATCCTCTTTTGAGGAACAAGTTTTCTTGTGCGACAGAAT
7	CAAGTTTGCCTCCTCTTTTGAGGAACAAGTTTTCTTGTTTTAGCGTCA
8	GACTGTAGCGTCCTCTTTTGAGGAACAAGTTTTCTTGTCGTTTTCATC
9	GGCATTTTCGTCCTCTTTTGAGGAACAAGTTTTCTTGTGTCATAGCCC
10	CCTTATTAGCTCCTCTTTTGAGGAACAAGTTTTCTTGTGTTTGCCATC
11	TTTTCATAATTCCTCTTTTGAGGAACAAGTTTTCTTGTCAAAATCACC
12	GGAACCAGAGCCACCACCGGAACCGCCTCCCTCAGAGCCGCCACCC

Table S4 Sequences of the oligonucleotides used to form DNA nanostructures at position A. The parts of supplementary strands that are complementary to corresponding customized staples are marked in blue.

AP	AGAAACCACCAGAAGGAGCGGAATTATCATCATA
AO2	CCTTTGCCCGAACGTTATTA
AO3	ATTTTAAAAGTTTGAGTAAC
AO4	ATTATCATTTTGCGGAACAA
A1T10	TTCGACAACTCGTATTAAATTTTTTTTTTT
A2T10	CCTTTGCCCGAACGTTATTATTTTTTTTTT
A3T10	ATTTTAAAAGTTTGAGTAACTTTTTTTTTT
A4T10	ATTATCATTTTGCGGAACAATTTTTTTTTT
A1T30	TTCGACAACTCGTATTAAATTTTTTTTTTTTTTTTTTTTTTTTTTTTTTT
A2T30	CCTTTGCCCGAACGTTATTATTTTTTTTTTTTTTTTTTTTTTTTTTTTTT
A3T30	ATTTTAAAAGTTTGAGTAACTTTTTTTTTTTTTTTTTTTTTTTTTTTTTT
A4T30	ATTATCATTTTGCGGAACAATTTTTTTTTTTTTTTTTTTTTTTTTTTTTT
A1C30	TTCGACAACTCGTATTAAATCCCCCCCCCCCCCCCCCCCCCCCCCCCCCC
A2C30	CCTTTGCCCGAACGTTATTACCCCCCCCCCCCCCCCCCCCCCCCCCCCCC
A3C30	ATTTTAAAAGTTTGAGTAACCCCCCCCCCCCCCCCCCCCCCCCCCCCCC
A4C30	CATTATCATTTTGCGGAACAACCCCCCCCCCCCCCCCCCCCCCCCCCCCCC
AD1	TTCGACAACTCGTATTAAATTGGATGTGTGCCATAGTGGATTGCGGCTGA
AD2	CCTTTGCCCGAACGTTATTATGGATGTGTGCCATAGTGGATTGCGGCTGA
AD3	ATTTTAAAAGTTTGAGTAACTGGATGTGTGCCATAGTGGATTGCGGCTGA
AD4	ATTATCATTTTGCGGAACAATGGATGTGTGCCATAGTGGATTGCGGCTGA
cAD	TCAGCCGCAATCCACTATGGCACACATCCA
AH1	TTCGACAACTGTGAGAATGAGACTTTTGTCTCATTCTCACCGTATTAAAT
AH2	CCTTTGCCCGGTGAGAATGAGACTTTTGTCTCATTCTCACAACGTTATTA
AH3	ATTTTAAAAGGTGAGAATGAGACTTTTGTCTCATTCTCACTTTGAGTAAC
AH4	ATTATCATTTTGTGAGAATGAGACTTTTGTCTCATTCTCACGCGGAACAA
AW1	TTCGACAACTCGTATTAAATGGATGTTAGTGGATGGCTGA
AW2	CCTTTGCCCGAACGTTATTAGGATGTTAGTGGATGGCTGA
AW3	ATTTTAAAAGTTTGAGTAACGGATGTTAGTGGATGGCTGA
AW4	ATTATCATTTTGCGGAACAAGGATGTTAGTGGATGGCTGA
HA4	GGCATCTAGCTACGAGGCTTTTGCCTCGTAGCTAGATGCCTCAGCCATCC ACTAACATCC

Table S5 Sequences of the oligonucleotides used to form DNA nanostructures at position B.

BP	CAAAATCATAGGTCTGAGAGACTACCTTTTTAAC
BO3	CTTAGATTAAGACGCTGAGA
BO4	AGAGTCAATAGTGAATTTAT
B1T15	AATTAATTTTCCCTTAGAATTTTTTTTTTTTTTTT
B2T15	CCTTGAAAACATAGCGATAGTTTTTTTTTTTTTTT
B3T15	CTTAGATTAAGACGCTGAGATTTTTTTTTTTTTTT
B4T15	AGAGTCAATAGTGAATTTATTTTTTTTTTTTTTTT
B1T30	AATTAATTTTCCCTTAGAATTTTTTTTTTTTTTTTTTTTTTTTTTTTTTT
B2T30	CCTTGAAAACATAGCGATAGTTTTTTTTTTTTTTTTTTTTTTTTTTTTTT
B3T30	CTTAGATTAAGACGCTGAGATTTTTTTTTTTTTTTTTTTTTTTTTTTTTT
B4T30	AGAGTCAATAGTGAATTTATTTTTTTTTTTTTTTTTTTTTTTTTTTTTTT
B1A30	AATTAATTTTCCCTTAGAATAAAAAAAAAAAAAAAAAAAAAAAAAAAAAA
B2A30	CCTTGAAAACATAGCGATAGAAAAAAAAAAAAAAAAAAAAAAAAAAAAAA
B3A30	CTTAGATTAAGACGCTGAGAAAAAAAAAAAAAAAAAAAAAAAAAAAAAAA
B4A30	AGAGTCAATAGTGAATTTATAAAAAAAAAAAAAAAAAAAAAAAAAAAAAA
BH1	AATTAATTTTCCCTTAGAATGTGAGAATGAGACTTTTGTCTCATTCTCAC
BH2	CCTTGAAAACATAGCGATAGGTGAGAATGAGACTTTTGTCTCATTCTCAC
BH3	CTTAGATTAAGACGCTGAGAGTGAGAATGAGACTTTTGTCTCATTCTCAC
BH4	AGAGTCAATAGTGAATTTATGTGAGAATGAGACTTTTGTCTCATTCTCAC
BW1	AATTAATTTTCCCTTAGAATTGAGGATGTGAGAATGAGAC
BW2	CCTTGAAAACATAGCGATAGTGAGGATGTGAGAATGAGAC
BW3	CTTAGATTAAGACGCTGAGATGAGGATGTGAGAATGAGAC
BW4	AGAGTCAATAGTGAATTTATTGAGGATGTGAGAATGAGAC
BR1	AATTAATTTTCCCTTAGAATGTGGTTACGACGAGATCTTGGTGGACTTGG
BR2	CCTTGAAAACATAGCGATAGGTGGTTACGACGAGATCTTGGTGGACTTGG
BR3	AGAGTCAATAGTGAATTTATGTGGTTACGACGAGATCTTGGTGGACTTGG
BR4	AGAGTCAATAGTGAATTTATGTGGTTACGACGAGATCTTGGTGGACTTGG
HB12	CGCTCCGATAGCTCTTTTTTTTTTTTGAGCTATCGGAGCGGTCTCATTCTCACATCCTCA
B20	GTCTCATTCTCACATCCTCA
B60	Phos-CTCAGCAGACTTTTTTTTTTTTTTTTTTGTCTCATTCTCACATCCTCATTATCGCCTATC
Sp	GAGTGTGTCTGCTGAGGATAGGCGAT
cS	ATCGCCTATCCTCAGCAGACACACTC

Table S6 Sequences of the oligonucleotides used to form DNA nanostructures at position C.

CP	CCAATCAATAATCGGCTGTCTTTCCTTATCATTC
CO4	AATTTACGAGCATGTAGAAA
C1T30	GAACGCGCCTGTTTATCAACTTTTTTTTTTTTTTTTTTTTTTTTTTTTTT
C2T30	AATAGATAAGTCCTGAACAATTTTTTTTTTTTTTTTTTTTTTTTTTTTTT
C3T30	GAAAAATAATATCCCATCCTTTTTTTTTTTTTTTTTTTTTTTTTTTTTTT
C4T30	AATTTACGAGCATGTAGAAATTTTTTTTTTTTTTTTTTTTTTTTTTTTTT
C1T20	GAACGCGCCTGTTTATCAACTTTTTTTTTTTTTTTTTTTT
C2T20	AATAGATAAGTCCTGAACAATTTTTTTTTTTTTTTTTTTT
C3T20	GAAAAATAATATCCCATCCTTTTTTTTTTTTTTTTTTTTT
C4T20	AATTTACGAGCATGTAGAAATTTTTTTTTTTTTTTTTTTT
CW1	GAACGCGCCTGTTTATCAACGTCCAGAACACACTCCTAAC
CW2	AATAGATAAGTCCTGAACAAGTCCAGAACACACTCCTAAC
CW3	GAAAAATAATATCCCATCCTGTCCAGAACACACTCCTAAC
CW4	AATTTACGAGCATGTAGAAAGTCCAGAACACACTCCTAAC
HC20	CGCTAAGCTGTTTTTTTTTTTTTTTTTTTTCAGCTTAGCG GTTAGGAGTGTGTTCTGGAC

Table S7 Sequences of the oligonucleotides used to form DNA nanostructures at position D.

DP	AAGAGCAAGAAACAATGAAATAGCAATAGCTATC
DO2	GGGTAATTGAGCGCTAATAT
DO3	CAGAGAGATAACCCACAAGA
DO4	ATTGAGTTAAGCCCAATAAT
D1T30	ACACCCTGAACAAAGTCAGATTTTTTTTTTTTTTTTTTTTTTTTTTTTTT
D2T30	GGGTAATTGAGCGCTAATATTTTTTTTTTTTTTTTTTTTTTTTTTTTTTT
D3T30	CAGAGAGATAACCCACAAGATTTTTTTTTTTTTTTTTTTTTTTTTTTTTT
D4T30	ATTGAGTTAAGCCCAATAATTTTTTTTTTT TTTTTTTTTTTTTTTTTTTT
D1G30	ACACCCTGAACAAAGTCAGAGGGGAGGGGAGGGGAGGGGAGGGGAGGGGA
D2G30	GGGTAATTGAGCGCTAATATGGGGAGGGGAGGGGAGGGGAGGGGAGGGGA
D3G30	CAGAGAGATAACCCACAAGAGGGGAGGGGAGGGGAGGGGAGGGGAGGGGA
D4G30	ATTGAGTTAAGCCCAATAATGGGGAGGGGAGGGGAGGGGAGGGGAGGGGA
DW1	ACACCCTGAACAAAGTCAGAGGCACAGCTATAATAACGCA
DW2	GGGTAATTGAGCGCTAATATGGCACAGCTATAATAACGCA
DW3	CAGAGAGATAACCCACAAGAGGCACAGCTATAATAACGCA
DW4	ATTGAGTTAAGCCCAATAATGGCACAGCTATAATAACGCA
HD28	CGCGCGTTTTTTTTTTTTTTTTTTTTTTTTTTTTTTCGCGCGTGCGTTATTATAGCTGTGCC
DWT10a	TTTTTTTTTTGTTGGTAGTGTGCGTTATTATAGCTGTGCC
DWT10b	CACTACCAACTTTTTTTTTT
D60	CTCAGCAGACTTTTTTTTTTTTTTTTTTTGCGTTATTATAGCTGTGCCTTATCGCCTATC
DE1	ACACCCTGAACAAAGTCAGACGACAAGCAGTGAGCTAGGTTGCGTTATTATAGCTGTGCC
DE2	ACCTAGCTCACTGCTTGTCGTCTGACTTTGTTCAGGGTGTGATCACTTGCTACAGTTGTG
DE3	CACAACTGTAGCAAGTGATC

S2 Supporting theories

S2.1 Principle of DNA carrier-based nanopore sensing

It is necessary to illustrate how our designs of DNA carrier can result in multi-level drops in the nanopore current trace. The current response of a nanopore sensor, usually called a resistive pulse, is induced when a translocation event temporarily blocks the pathway of ions through the nanopore thereby changing the nanopore resistance. The side containing analyte molecules is defined as the *cis* side of the nanopore, and the side at which the analytes finally arrive after translocation is the *trans* side. In general, the nanopore resistance can be divided into that of the open pore region R_{pore} and that of the access region R_{acc} (Figure S11a). For a conical nanopore, the inherent resistance generated by the movement of ion molecules in solution through the nanometric aperture can be written as below[1].

$$R = R_{pore} + R_{acc} = \rho\frac{4L}{\pi D_{trans} D_{cis}} + \rho(\frac{1}{2D_{trans}} + \frac{1}{2D_{cis}})$$

where ρ is the resistivity of the electrolyte solution, L is the length of the nanopore, D_{trans} and D_{cis} are respectively the diameters of the *trans* opening and the *cis* opening of the nanopore. If we simplify the local analyte to a nanosphere with a diameter of d, and x is the distance of its center from the *cis* opening of the nanopore, then the change in nanopore resistance due to its presence can be calculated as the following formula[1].

$$\Delta R = \Delta R_{pore} + \Delta R_{acc} = \frac{4\rho d^3}{\pi D_{cis}^4} + \rho\{\int_{-x}^{\frac{d}{2}} \frac{dz}{\pi\left[\frac{D_{cis}}{2} + (z+x)\frac{4}{\pi}\right]^2 - \pi\left(\frac{d^2}{4} - z^2\right)} - \int_{-x}^{\frac{d}{2}} \frac{dz}{\pi\left[\frac{D_{cis}}{2} + (z+x)\frac{4}{\pi}\right]^2}\}$$

Accordingly, for a particular nanopore, the diameter d and position x of the analyte are essential parameters that affect ΔR under constant solution conditions. As shown in Figure S8b, when the DNA carrier backbone enters the access region, d is defined by the diameter of the characteristic double-helix structure of ds DNA and remains basically the same. The nanopore current drops as x becomes smaller until the backbone occupies the whole response area and the current trace arrives at the first-level platform. At the point when the secondary DNA nanostructure on the carrier reaches the access region, d significantly increases, resulting in a further decrease of nanopore current. The current drops to its lowest point when the synergistic effect of d and x reaches its maximum. Then as the DNA carrier comes out of the response area, the nanopore resistance changes in reverse until the current trace returns to the original baseline. Based on this principle, the magnitude, duration, and frequency of the multi-level resistance pulses can reveal much useful information about properties of the DNA carrier and the secondary nanostructures on it, such as size, shape, charge, and many more.

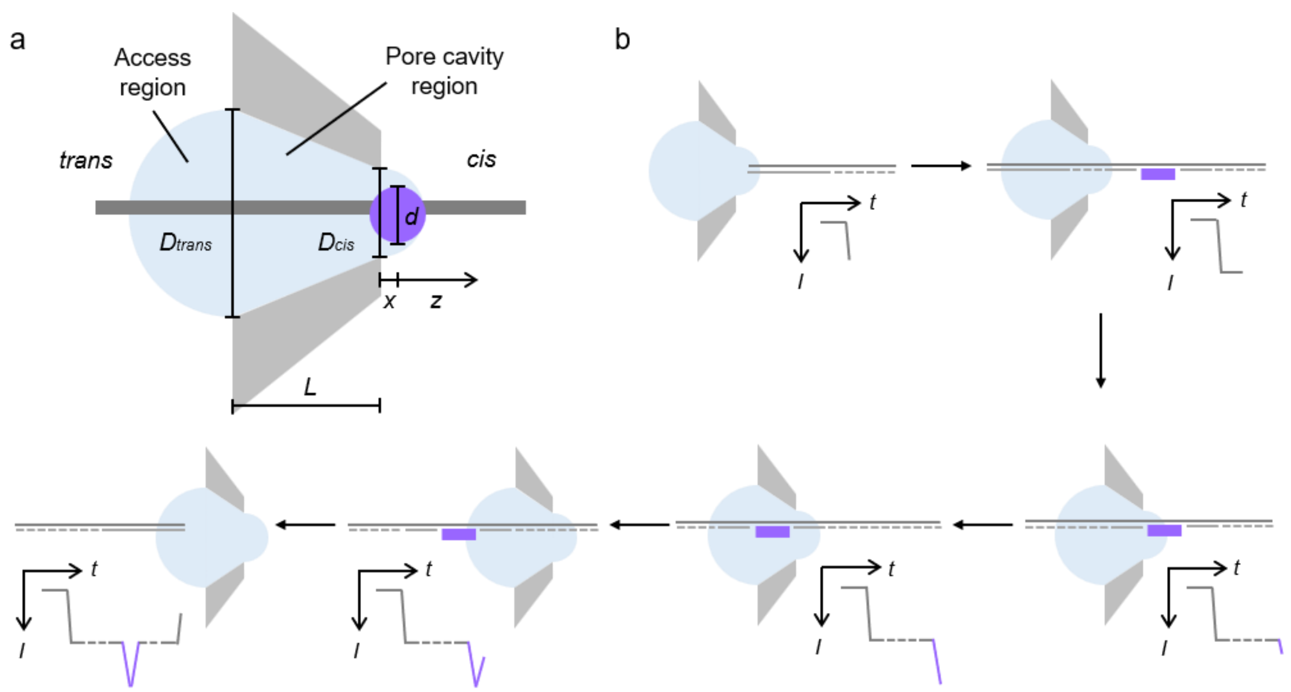

Figure S11 Principle of the generation of multi-level nanopore current signals. (**a**) Schematic illustration of a DNA carrier translocating through the access region and the pore cavity region of the nanopore. (**b**) Schematic of the detailed process of which the current trace is in different degree affected as a carrier is driven through the nanopore. The entering of the bare DNA scaffold into the sensitive regions produces the first-level current drop. When the secondary structure starts to pass through the nanopore tip, the second-level current drop appears. When the secondary structure arrives at the center of the sensitive region and occupies the largest possible volume, the current trace reaches the bottom. When the secondary structure begins to flow out of the sensitive tip regions, the current goes back.

S2.2 Fittings of normalized peak depth and the number and length of poly-dT overhangs

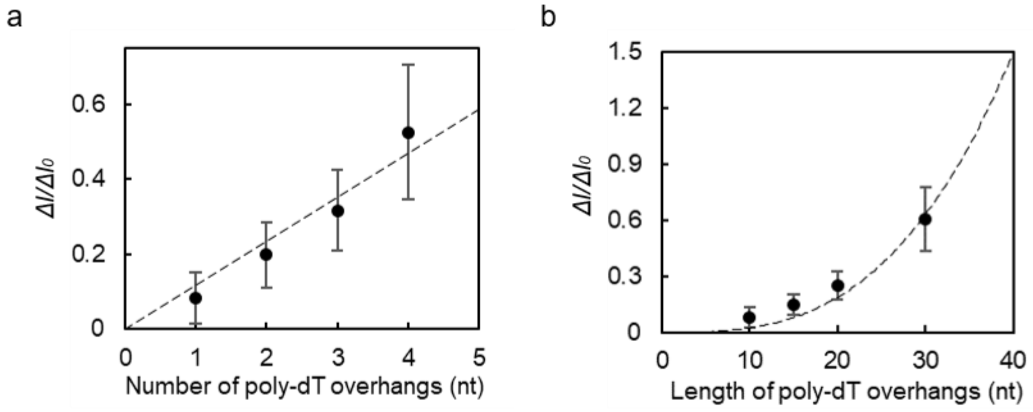

Figure S12 (**a**) Linear fitting of the average peak depth and the number of poly-dT overhangs in the structures on Carrier 1. (**b**) Cubic fitting of the average peak depth and the length of poly-dT overhangs in the structures on Carrier 2.

In Figure S9a, we show $\Delta I/\Delta I_0$ as a function of the number of poly-dT overhangs on Carrier 1 and find a linear

relationship, as expected. We force the trendline to cross the origin of coordinates because no signal is expected when there's no nanostructure. In contrast, a cubic polynomial can fit the relationship between peak depth and the length of poly-dT overhangs on Carrier 2 (Figure S12b). One explanation is that the overhangs are with one end anchored to the carrier backbone and the other end moving flexibly in the solution in all directions, so the maximum range of space that an overhang may occupy in the solution around the anchor point can be seen as a ball with the length of the overhang as the radius. The success of both the linear (Figure S12a) and cubic (Figure S12b) fits demonstrate that nanopore current signals are related to the volume of secondary nanostructures in DNA carriers. This opens the pathway to the systematic study of more complex DNA sequences.

S2.3 Worm-like chain (WLC) model

The WLC model[2-4] describes polymers whose successive segments are orientationally cooperative and the flexibility is brought by fluctuations of the contour rather than large-angle bond rotations. For a polymer of maximum length L_0 (for dsDNA, $L_0 = 0.34\, nm \times number\ of\ bp$[5]; for ssDNA, $L_0 = 0.43\, nm \times number\ of\ nt$[6]), parametrize its path as $s \in (0, L_0)$. Allow $\vec{r}(s)$ to be the position vector along the DNA chain at point s (Figure S13), then the energy associated with the bending of the DNA can be written as:

$$E = \frac{1}{2} k_{\boldsymbol{B}} T \int_0^{L_0} P \cdot \left(\frac{\partial^2 \vec{r}(s)}{\partial s^2}\right)^2 ds$$

where $k_{\boldsymbol{B}}$ is the Boltzmann constant, T is the absolute temperature, and P is the polymer's characteristic persistent length which is usually within a few orders of magnitude of the chain length (P of 30 nm and 2.4 nm are estimated for dsDNA and ssDNA in this work[7]).

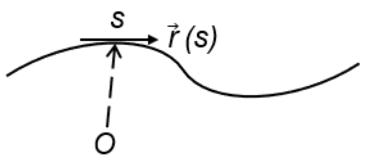

Figure S13 Illustration of the WLC model, with position s and its unit vector $\vec{r}(s)$ as shown.

At finite temperatures, a coiled and random configuration of the undisturbed polymer is often caused by thermal fluctuations, so the end-to-end distance (L_E) of the polymer is significantly shorter than L_0. When $L_0 < P$, the average value of L_E under minimum E can be calculated as:

$$< |L_E|^2 > = 2PL_0 \left[1 - \frac{P}{L_0}\left(1 - e^{-\frac{L_0}{P}}\right)\right]$$

S3 Supplementary experiments

S3.1 Influence of attachment position on nanopore current signal

Our design has four functional attachment positions along the carrier. Since the sequence of M13 is different at these four positions, the staples and overhangs designed accordingly also have different sequences and thus have different tendency to self-fold or interact with each other. In order to make a more convincing comparison of the nanopore current signals generated by different structures bound to different positions on the same carrier, a preliminary experiment studying the influence of attachment position on the significance of ion current drop is required. On Carrier 10, there are four repeats of 30 nt poly-dT overhangs at position A, B, C, and D respectively. Nanopore measurement result is shown below.

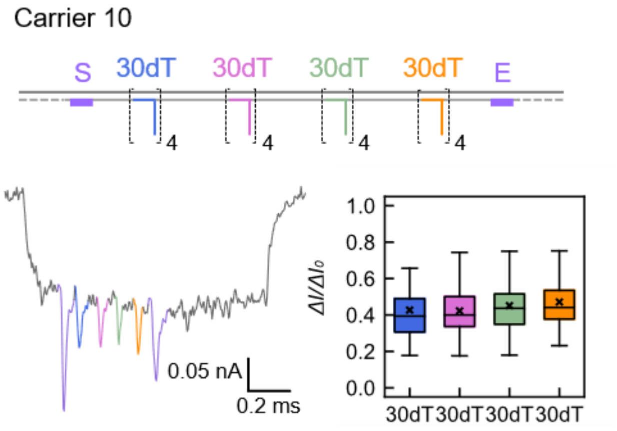

Figure S14 Schematic and nanopore measurement result of Carrier 10 with four repeats of 30 nt poly-dT overhangs at position A, B, C, and D respectively.

From the boxplot, we can see that although the peak depth of 4x30 nt poly-dT at the four positions is a little bit different from each other, the difference is not huge compared to the difference resulting from the different structures we designed on the same carrier, thus can be neglected when we do the analysis. However, we admit that in cases where we need to do careful comparison, it is necessary to take into account the influence of staple sequences at different positions, and make better choices at the design stage.

S3.2 More repeats or extended length

It may be interested to some readers If we bind a long structure with the same length at one position, for example replacing 4x20 bp dsDNA with 1x80 bp dsDNA, would it create the same signal or even higher due to the flexibility. Accordingly, we synthesized Carrier 11 and got the following result (Figure S15). From the boxplot, we can see that the 80 bp dsDNA generated a similar signal to the 4x20 bp structure on average, especially the range of peak depth of these two structures is almost the same. But the average peak depth of the 80 bp dsDNA is smaller than the 4x20 bp structure. We calculated the end-to-end lengths (L_E) of 80 bp and 20 bp dsDNA and got 23.64 nm and 6.55 nm respectively. The L_E of 80 bp dsDNA is a bit smaller than four times the L_E of 20 bp dsDNA (26.20 nm), so it is reasonable to think that the 80 bp dsDNA overhang is not as extended and flexible as four repeats of 20 bp dsDNA, thus leading to slightly weaker nanopore signals. Another possible explanation is that the length of 80 bp dsDNA is larger than the diameter of our glass nanopore (about 10 nm), and long overhangs are more likely to lie down when the carrier translocates through the nanopore. From the results of Carrier 6, such "lying down" could also result in lower peak depth.

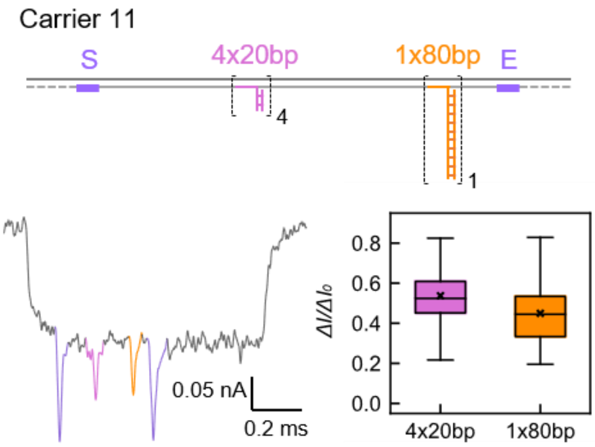

Figure S15 Schematic and nanopore measurement result of Carrier 11 with four repeats of 20 bp dsDNA overhangs at position B, and a single 80 bp dsDNA overhang at position D.

S3.3 Dependence on sequence

Besides Carrier 3 containing poly-dC and poly-dT structures, and Carrier 4 containing poly-dA and dG-rich structures, a third carrier is required to make all categories of nucleotides interrelated. From the nanopore measurement result of Carrier 12 with poly-dT and dG-rich structures (Figure S16a), we find a wider range of peak depths resulted from dG-rich than poly-dT though the current signals they generate are both generally more intense than poly-dA and poly-dC, as indicated in the results of Carrier 3 and Carrier 4. When we scrutinize the events from Carrier 12 one by one, we find some event traces with considerably high or low dG-rich peaks, while the peak depths of poly-dT are always within rational bounds (Figure S16b). This helps verify our hypothesis that dG-rich overhangs can interact with each other and form unexpected complex 3D structures under our experimental conditions. Therefore, they are not as ideal as poly-dT strands as candidates for ssDNA nanopore study. Scatterplots of peak depth in relation to the location of these secondary structures on Carriers 3, 4, and 12 are presented in Figure S16c as adminicular evidence. We also demonstrate the better flexibility of poly-dT than randomly sequenced overhangs of the same length on Carrier 13 (Figure S17).

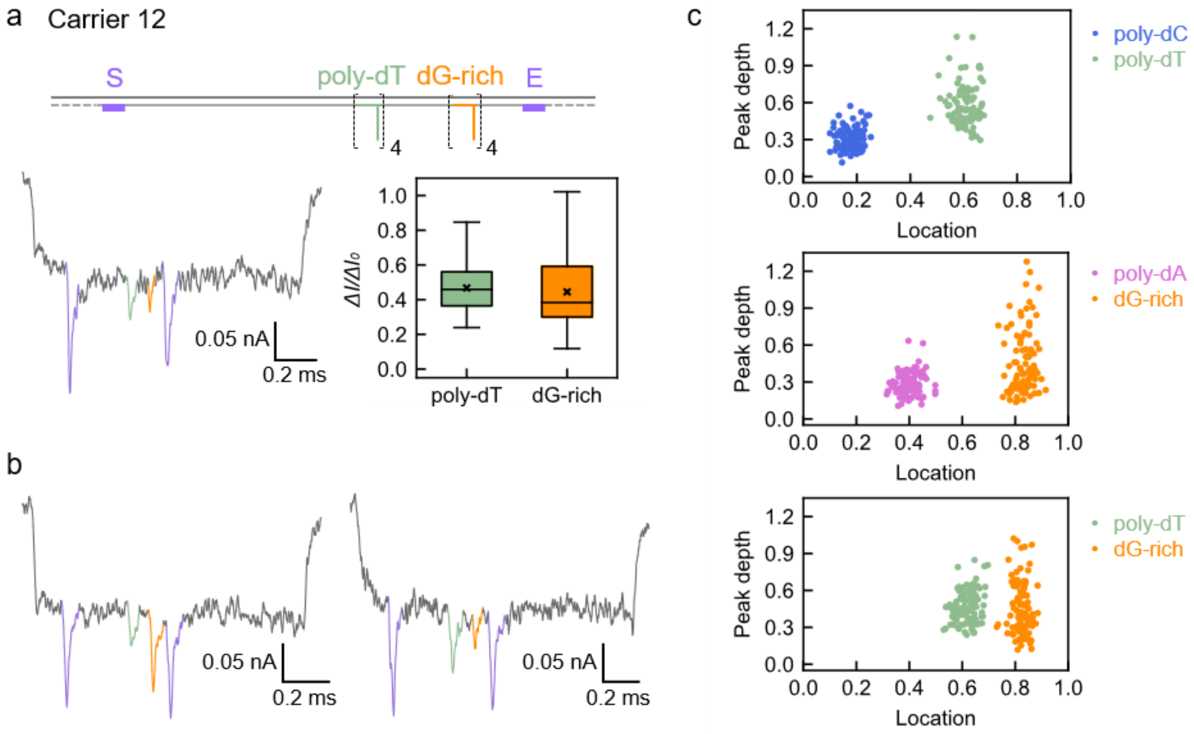

Figure S16 Supplementary studies on the dependence of nanopore current signals on DNA sequence. (**a**) Schematic and nanopore measurement result of Carrier 12 with 30 nt poly-dT and dG-rich overhangs at positions C and D, respectively. (**b**) Representative unfolded events of Carrier 10 with especially high or low dG-Rich peaks after baseline correction. (**c**) Scatterplots of peak depth in relation to the location of the secondary structures on Carriers 3, 4 and 12.

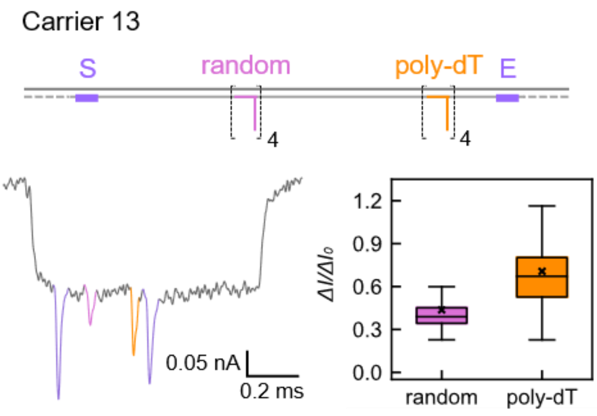

Figure S17 Schematic and nanopore measurement result of Carrier 13 with four repeats of 30 nt random-sequenced overhangs at position B, and four repeats of 30 nt poly-dT at position D.

S3.4 Dependence on nanopore size

Although we fabricated the nanopores following a consistent protocol, it was quite hard for us to get ones with exactly the same size. To investigate whether the size of nanopore might influence the current signals we got, we made both smaller and larger nanopores by setting the HEAT of the puller 20 higher or lower (than 470) while keeping all the other parameters unchanged, and measured Carrier 5 with them. From results shown in Figure S18, we find that the relative significance of the three DNA nanostructures on Carrier 5 remains the same as the size of the nanopore

increases, but the absolute current drops they bring about gradually decrease since these structures appear smaller compared to the cross-sectional area of a larger nanopore. The almost overlapping trend lines of the two identical reference structures in Figure S18d demonstrate the reliability of our measurement results.

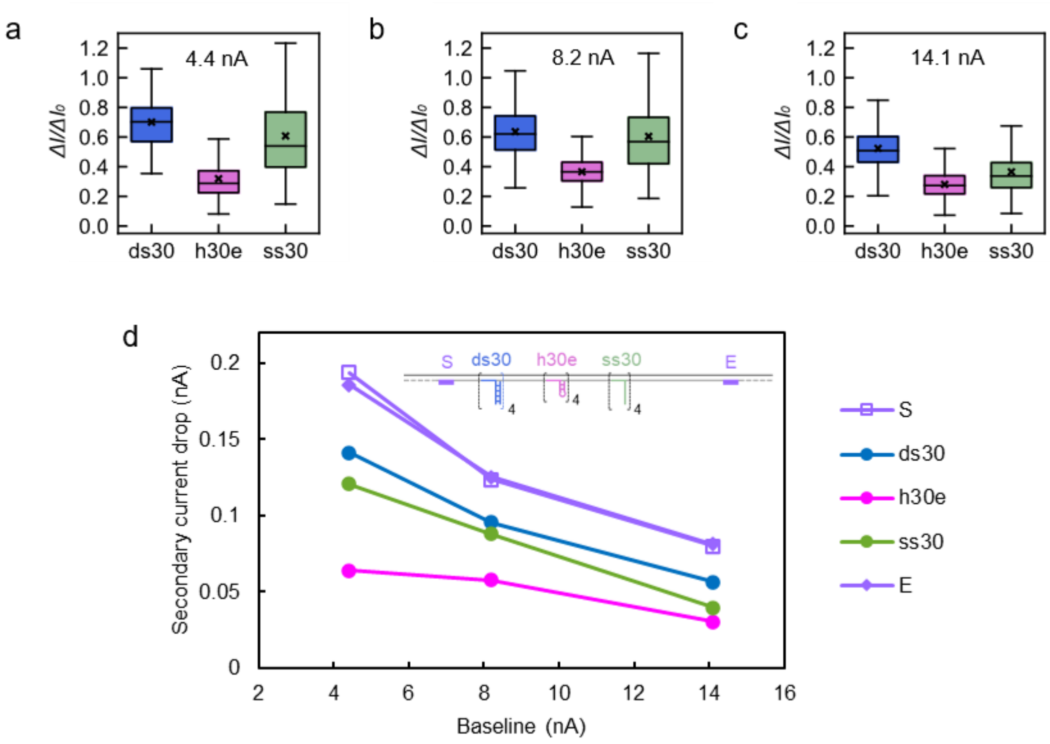

Figure S18 Statistical boxplots of Carrier 5 measurement results by nanopores with a current baseline of around (**a**) 4.4 nA, (**b**) 8.2 nA, and (**c**) 14.1 nA. (**d**) Line chart of absolute secondary current drops brought about by the nanostructures on Carrier 5 in relation to the current baselines of different nanopores. Average values are used.

S4 Experiment details

S4.1 Design and synthesis of DNA carriers

All the DNA oligonucleotides were purchased from Integrated DNA Technologies, Inc. (IDT). Circular M13mp18 DNA was purchased from New England Biolabs. Linear ssDNA scaffold was obtained by hybridizing

45 μL M13mp18 DNA (224 ng/μL)
8 μL 10 × New England Biolabs cutsmart buffer
2 μL oligonucleotide (100 μM) with sequence5'-TCTAGAGGATCCCCGGGTACCGAGCTCGAATTCGTAATC-3'
23 μL deionized water

at 65°C followed by a linear cooling ramp to 25°C over 40 minutes. Purification was performed with Macherey-Nagel NucleoSpin Gel and a PCR clean-up kit (Fisher Scientific) immediately after cutting the hybrid with 1 μL BamHI-HF and 1 μL EcoRI-HF enzymes (New England Biolabs, 100 units/μL) at 37°C for an hour.

A total of 190 oligonucleotides (referred to as staple 1 to staple 190 in order) complementary to the entire 7228 nt scaffold were designed as in our previous work[8]. DNA nanostructures were formed at well-defined positions by selectively replacing individual oligonucleotides. In this work, staples 26-32 and staples 96-102 were respectively replaced with a "reference" structure, each consisting of 11 dumbbells. Since the two reference structures were not at the same distance from the ends of the M13mp18 scaffold (the one nearer to the end of the carrier is called reference_start (S), and the one in the middle part of the chain is called reference_end (E)), we could easily identify the direction from which a DNA carrier passed through the nanopore by analyzing the relative locations of secondary drops in the current trace. Another four binding areas were specified for functional nanostructure designs: staples 42-44 (position A), staples 56-58 (position B), staples 70-72 (position C), and staples 84-86 (position D). Each functional position, as long as it played a part, was occupied by four customized strands and one supplementary oligonucleotide (staple AP, BP, CP, and DP at positions A, B, C, and D, respectively). Otherwise, the original complementary staples were added. The backbone of each carrier was formed by mixing

8 μL cut M13mp18 DNA (100 nM)
4 μL staple mix (each staple 1000 nM)
2.8 μL TM buffer (10 mM Tris-HCl, 10 mM $MgCl_2$, pH=7.5)
1 μL $MgCl_2$ (100 mM)
24.2 μL deionized water

followed by heating to 70°C and then linearly cooling to 25°C in a thermocycler over 50 minutes. The staples were at 5 times excess to the scaffold.

For carriers containing double-stranded structures, 16 μL of 1 μM additional strands other than the customized staples (cAD in Carrier 5; cB20 in Carrier 11; circular B60 and D60 in Carrier 8) were added to the annealed mixture and incubated at room temperature for an hour. For carriers containing stem-loop or junction structures, the additional strands (HA4, HB12, HC20, and HD28 in Carrier 7; HC20 and (DWT10a+DWT10b) in Carrier 8; DE1, DE2, and DE3 in Carrier 11) were individually heated to 88°C followed by a linear cooling ramp to 25°C over 40 minutes before being added to the carrier solution. Sketched designs of carriers 1 to 10 and detailed staple sets of each carrier are given in S3. After the annealing and incubation procedures, excess oligonucleotides were then removed with washing buffer (10 mM Tris-HCl, 0.5 mM $MgCl_2$, pH=8) by centrifuging at 9000g for 10 minutes using Amicon Ultra 100kDa filters for successive two times. Typically, we could collect about 30 μL solution of purified DNA carriers after washing, which was then quantified with NanoDrop 2000 spectrophotometer. All the carrier solutions

were frozen in a refrigerator at -20°C for later use.

S4.2 Preparation of circular DNA

The hybrid (BS) of linear 5'-phosphorylated oligonucleotide (B60) and its corresponding splint strand (Sp) was obtained by mixing

10 µL B60 (100 µM)
15 µL Sp (100 µM)
75 µL TM buffer

and then heating to 95°C for 5 minutes, followed by slowly cooling down to room temperature. The ligation was performed by mixing

25 µL BS (10 µM)
2 µL T4 ligase (New England Biolabs, 400 units/µL)
5 µL 10 × ligase buffer (New England Biolabs)
18 µL deionized water

and incubating at 16°C for 16 h. Then, the ligase was inactivated by heating at 65°C for 10 minutes, giving us the BL solution. After curing the mixture of 36 µL BL (5 µM) and 3.24 µL cS (strand complementary to Sp, 100µM) at room temperature for 30 minutes to remove Sp from the circularized B60, the remaining linear DNA templates and short oligonucleotides were digested by mixing

36 µL BL (5 µM) + 3.24 µL cS (100µM)
1.5 µL Exonuclease I (New England Biolabs, 20 units/µL)
5 µL 10 × Exonuclease I buffer (New England Biolabs)
4.26 µL deionized water

and incubating at 37°C for 30 minutes, followed by heating at 60°C for 15 minutes, while circular B60 was left intact in the resulting BLE solution. BSC solution and BLC solution were also prepared by mixing

2 µL BS (10 µM) or 4 µL BL (5 µM)
1.8 µL cS (20 µM)
6.2 µL TM or 4.2 µL TM

and incubating at 37°C for 30 minutes.

The purification of circular B60 was realized by 15% native polyacrylamide gel electrophoresis (PAGE) in lane 7 to lane 10. Lane 1 was added with a low molecular weight DNA ladder as a control. Lane 2 to lane 6 were added with different intermediate products of circular DNA preparation to demonstrate the successful synthesis of circular B60. Details are listed in the below table. The electrophoresis was conducted in 1 × TBE buffer (pH=8.0) with 10 mM $MgCl_2$ at a constant voltage of 110V (10 V/cm) for one hour. Then the gel was stained by GelRed for 15 minutes before visualization on a UV transilluminator. After cutting the corresponding bands out of lanes 7 to 10 of the gel with a razor blade under a UV light, the slices were chopped into fine pieces and transferred into a 1.5 mL microcentrifuge tube. TM buffer was added to the tube until it was just above the gel and kept at room temperature overnight to elute circular B60. The supernatant was collected using a pipette and quantified with NanoDrop 2000 spectrophotometer.

Table S8 The detailed recipe of each lane in 15% native PAGE analysis and purification.

	Analyte	TM buffer	6 × loading buffer
Lane 1	0.5 μL DNA ladder	9.5 μL	2 μL
Lane 2	1 μL B60 (20 μM)	9 μL	2 μL
Lane 3	2 μL BS (10 μM)	8 μL	2 μL
Lane 4	10 μL BSC (2 μM)	0	2 μL
Lane 5	4 μL BL (5 μM)	6 μL	2 μL
Lane 6	10 μL BLC (2 μM)	0	2 μL
Lane 7	5.6 μL BLE (3.6 μM)	4.4 μL	2 μL
Lane 8	10 μL BLE (3.6 μM)	0	2 μL
Lane 9	10 μL BLE (3.6 μM)	0	2 μL
Lane 10	10 μL BLE (3.6 μM)	0	2 μL

S4.3 Nanopore fabrication

Glass nanopores used in this work were fabricated by laser-assisted pulling (P-2000, Sutter Instrument) of quartz capillaries (outer diameter 0.5 mm and inner diameter 0.2 mm, Sutter Instrument). In this process, the small section of glass in the laser focus became malleable while a force was applied to both ends of the capillary in opposite directions until the capillary broke into two parts, each with a conically shaped tip. With tuned pulling parameters, nanoscale holes could be formed at the tip. Then the capillaries with a nanopore at one end were cut into a suitable length and placed in a 16-channel polydimethylsiloxane (PDMS) chip. The nanopore tips lay towards the central reservoir (cis), while the outer chambers (trans) of the chip housed the blunt sides of capillaries. Glass slides were used as the base to hold each PDMS chip after treatment in a plasma cleaner (Femto, Diener electronic) for 15 seconds. Before using the chips for measurements, they were placed in a plasma cleaner for three minutes to make their surface hydrophilic, followed by immediate pipetting of 4 M LiCl buffer (in 1 × TE buffer, pH 9.0) into all chambers. Two Ag/AgCl electrodes prepared by curing 1-mm Ag wires in a 10% solution of NaClO were separately inserted into the central reservoir and the outer chamber to create an electrical circuit across the nanopore. The one connecting the central reservoir served as the ground, while the other could be moved between outer chambers to address different nanopores. Current-voltage characteristic curves from -600 mV to 600 mV were recorded to indicate the estimated sizes of nanopores. Those with a maximum current of around 10 nA and a root-mean-square (RMS) noise below 7.5 pA were selected for further measurements. Based on a simplified equation for conical nanopores[9]:

$$d = \frac{2I}{\pi \sigma U \tan\theta}$$

where the electrical conductivity σ of 4 M LiCl solution is approximated to 15 S/m[10] in this work, and the inner taper angle θ is around 4°, we had the diameters of nanopores d (nm) ≈ $I_{600\,mV}$ (nA). Parameters of all the nanopores used in this work are listed in Table S9.

Table S9 Diameters and root-mean-square (RMS) noises of the nanopores used to measure each carrier.

	d (nm) ≈ $I_{600\,mV}$ (nA)	RMS (pA)
Carrier 1	10.1	6.4
Carrier 2	12.6	6.8
Carrier 3	9.9	6.3
Carrier 4	10.1	7.2
Carrier 5	4.4	5.9
	8.2	6.5
	14.1	7.1
Carrier 6	8.1	6.5
Carrier 7	10.2	6.5
Carrier 8	10.7	6.4

Carrier 9	11.7	6.5
Carrier 10	9.0	6.4
Carrier 11	8.7	6.2
Carrier 12	6.3	5.9
Carrier 13	8.5	6.2

S4.4 Nanopore measurement

Before nanopore measurement, carrier samples were unfrozen at room temperature and diluted to 1 nM with TM buffer, then diluted to 0.125 nM with 4 M LiCl buffer. Once functional nanopores had been identified following S4.3, the LiCl buffer in the central reservoir of the chip was replaced with prepared samples. A positive voltage of 600 mV was then applied to the outer chamber to drive negatively charged carrier molecules through the nanopore, creating characteristically transient changes in the ionic current trace. The signal was amplified by an Axopatch 200B patch clamp amplifier (Axon Instruments) with a 100 kHz sampling rate and filtered by a low-pass Bessel filter (Model 900CT, Frequency Devices) at 50 kHz. The conversion from analog to digital signal was realized by a data acquisition card (DAQ card, PCIe-6251 or PCIe-6351, National Instruments) with 16-bit resolution. Data analysis was performed with LabVIEW software developed by senior students in our group[8] and self-written python programs.

Folded events, as exemplified in Figure S19a, b, were left out to avoid additional affecting factors. From the current trace of unfolded events, as instantiated in Figure S19c, d, the six peaks indicating two references and positions A to D could be easily discriminated according to the distances between peaks and the ends of current drops. However, a small slope was often observed at the baseline, possibly due to the slight change in buffer concentrations as the measurement was carried out. Additionally, the absolute values of the first-level plateau and secondary drops were found somewhat related to the nanopore size. Hence in this work, the current baseline of each measurement was linearly fitted, and the slope was corrected (Figure S19e, f) before any further analysis. The depths and locations of the peaks were formalized by

$$Peak\ depth = \frac{\Delta I}{\Delta I_0}$$

$$Location = \frac{\Delta t}{\Delta t_0}$$

where ΔI_0 refers to the average value of the first-level current drop, which was mostly within the range of 0.1-0.2 nA. ΔI refers to the difference between the minimum current output within a second drop and ΔI_0. Δt_0 refers to the time scale between the two peaks generated by reference structures, typically lasting for 0.8-1.6 ms. Δt refers to the interval between any target peak and the peak suggesting reference S, namely the one nearer to the end of the DNA carrier.

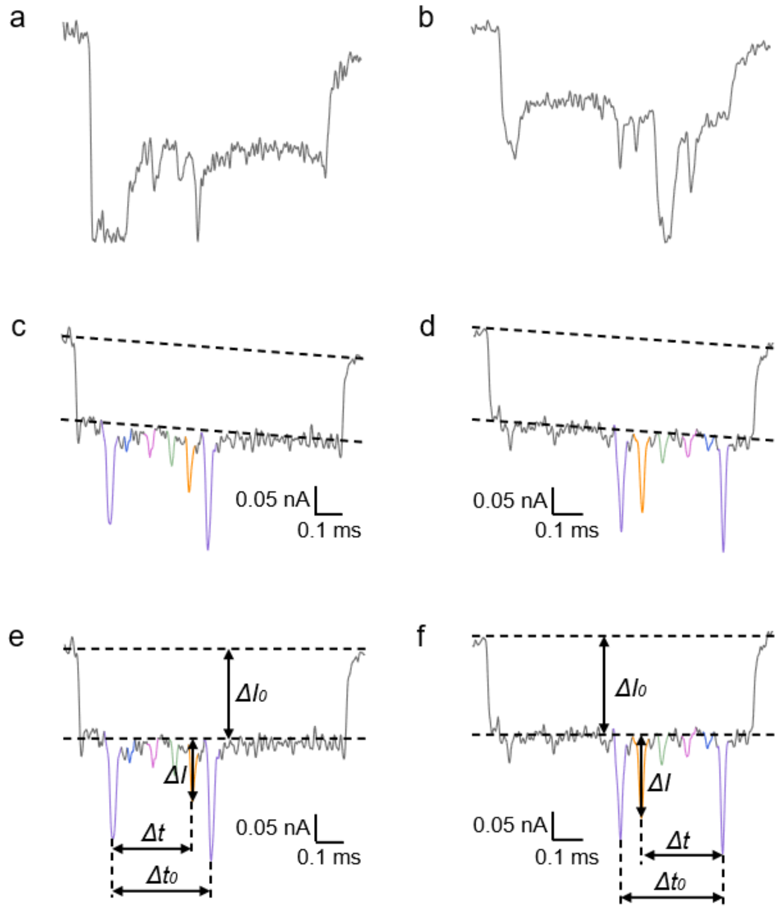

Figure S19 Current trace of (**a, b**) representative folded events, (**c, d**) representative unfolded events before correction of the baseline slope, and (**e, f**) the same unfolded events after slope correction.

Reference

(1) Tang, W.; Fried, J. P.; Tilley, R. D.; Gooding, J. J. Understanding and modelling the magnitude of the change in current of nanopore sensors. *Chem. Soc. Rev.* **2022**, *51*, 5757-5776.

(2) Muthukumar, M. *Polymer translocation*. CRC press, **2016**.

(3) Doi, M.; Edwards, S. F.; Edwards, S. F. *The theory of polymer dynamics*. Oxford University Press, **1988**.

(4) Rubinstein, M.; Colby, R. H. *Polymer physics*. Oxford University Press, **2003**, Vol. 23.

(5) Watson, J. D.; Crick, F. H. Molecular structure of nucleic acids: a structure for deoxyribose nucleic acid. *Nature* **1953**, *171*, 737-738.

(6) Amarasekara, C. A.; Athapattu, U. S.; Rathnayaka, C.; Choi, J.; Park, S.; Soper, S. A. Open-tubular nanoelectrochromatography (OT-NEC): gel-free separation of single stranded DNAs (ssDNAs) in thermoplastic nanochannels. *Electrophoresis* **2020**, *41*, 1627-1640.

(7) Bell, N. A. W.; Chen, K.; Ghosal, S.; Ricci, M.; Keyser, U. F. Asymmetric dynamics of DNA entering and exiting a strongly confining nanopore. *Nat. Commun.* **2017**, *8*, 380.

(8) Bell, N. A. W.; Keyser, U. F. Digitally encoded DNA nanostructures for multiplexed, single-molecule protein sensing with nanopores. *Nat. Nanotechnol.* **2016**, *11*, 645-651.

(9) Bell, N. A. W. *DNA origami nanopores and single molecule transport through nanocapillaries*, University of Cambridge, **2014**.

(10) Wu, X.; Gong, Y.; Xu, S.; Yan, Z.; Zhang, X.; Yang, S. Electrical conductivity of lithium chloride, lithium bromide, and lithium iodide electrolytes in methanol, water, and their binary mixtures. *J. Chem. Eng. Data* **2019**, *64*, 4319-4329..
